# Supplementary material for: Protein arginine methyltransferase 5 sustains Tip60-EP400 complex via SRSF1 in Merkel cell carcinoma
Source: Life Sci Alliance. 2025 Aug 22;8(11):e202503316. doi: 10.26508/lsa.202503316 (PMC12373721; doi:10.26508/lsa.202503316)
Supplement: Supplementary file 1 [file LSA-2025-03316_Supplemental_Data_1.docx]

# Replicate Summary for Western Blots

## Figure 1B and Figure EV1C

- Figure 1B and Figure EV1C represent similar experiments using two different PRMT5 inhibitors (JNJ-64619178 and LLY-283, respectively) with the same set of Western blots (SDMA, ADMA, MMA, GAPDH). Although not identical replicates, these experiments are complementary and reinforce each other’s conclusions.

## Figure 1C and Figure 1D

- Tip60 blot: three replicates
- SDMA blot: two replicates
- ADMA blot: two replicates
- Vinculin blot: four replicates

## Figure 1D

- EP400: one biological replicate; MAX IP and EP400 IP serve as two technical replicates.
- MAX: one biological replicate.

## Figure 5A

- Tip60: three replicates
- SDMA: two replicates
- HA (SRSF1): two replicates
- GAPDH: two replicates
- Vinculin: two replicates

## Figure 5B

- γ‑H2AX: two replicates
- PRMT5: two replicates
- Tip60: two replicates
- Vinculin: two replicates
- ADMA: two replicates
- EP400: two replicates
- SDMA: two replicates

## Figure 5C

- HA (SRSF1): two replicates
- YTHDC1: two replicates

## Figure EV1B

- SDMA, ADMA, MMA, GAPDH: one biological replicate each. The three different PRMT5 inhibitor concentrations (12 nM, 111 nM, 1000 nM), applied across four time points (1, 2, 3, and 4 days), serve as technical replicates.

## Figure EV1F

- ADMA: four replicates
- SDMA: two replicates
- MMA: two replicates
- GAPDH: two replicates

## Figure EV1G and Figure EV1H

- EV1G (12 nM PRMT5 inhibitor; H4K5ac, H4K12ac, H4K16ac, H3) and EV1H (0.3 nM PRMT5 inhibitor; H2A.Zac, H4K16ac, SDMA, H3) are similar experiments performed under different inhibitor concentrations and with partially overlapping targets. Each figure represents one biological replicate.
