# Supplementary material for: Protein arginine methyltransferase 5 sustains Tip60-EP400 complex via SRSF1 in Merkel cell carcinoma
Source: Life Sci Alliance. 2025 Aug 22;8(11):e202503316. doi: 10.26508/lsa.202503316 (PMC12373721; doi:10.26508/lsa.202503316)
Supplement: Supplementary file 2 [file LSA-2025-03316_SdataF1_F5_FS1.pptx]

## Slide 1
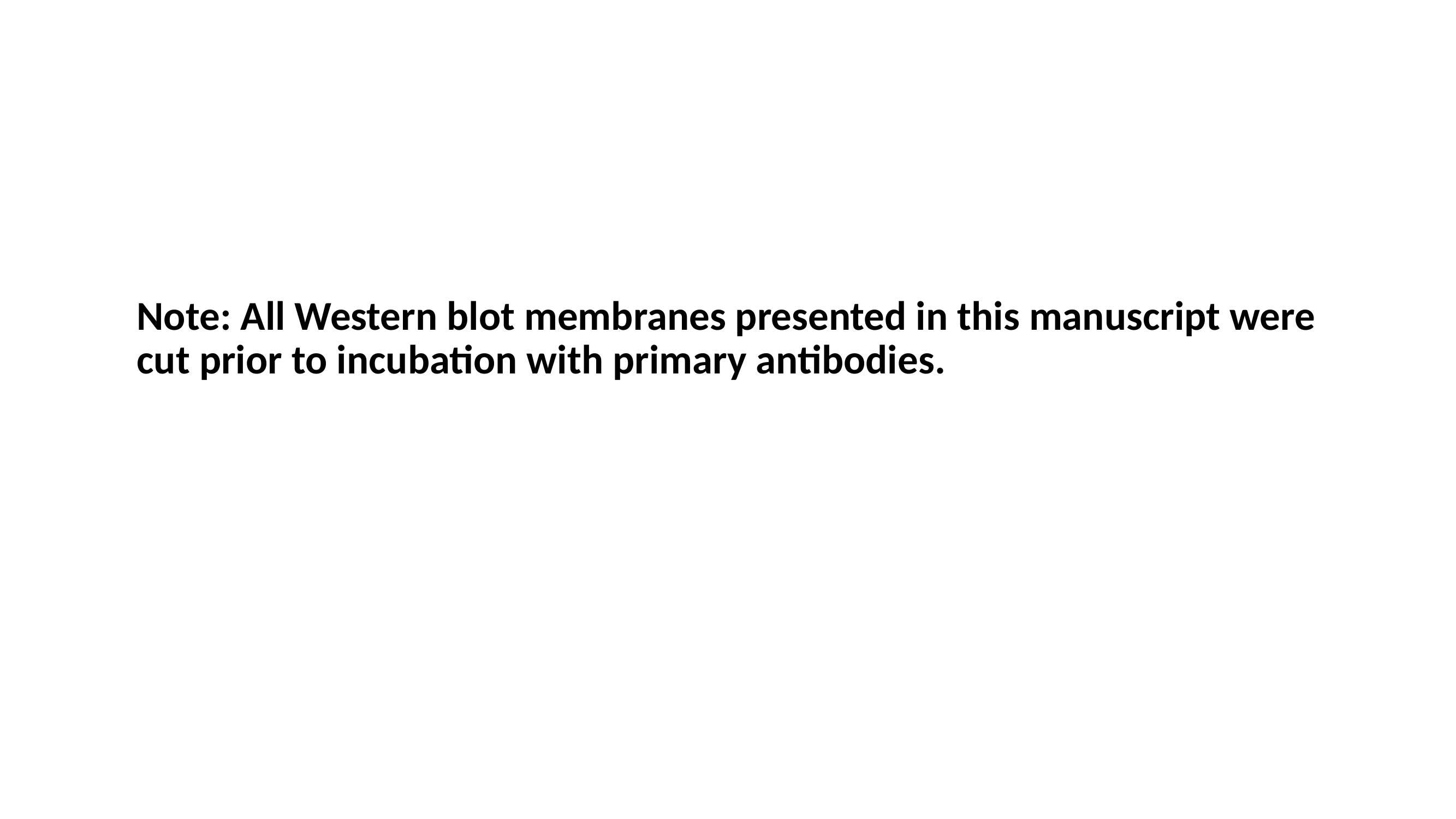

Note: All Western blot membranes presented in this manuscript were cut prior to incubation with primary antibodies.

## Slide 2
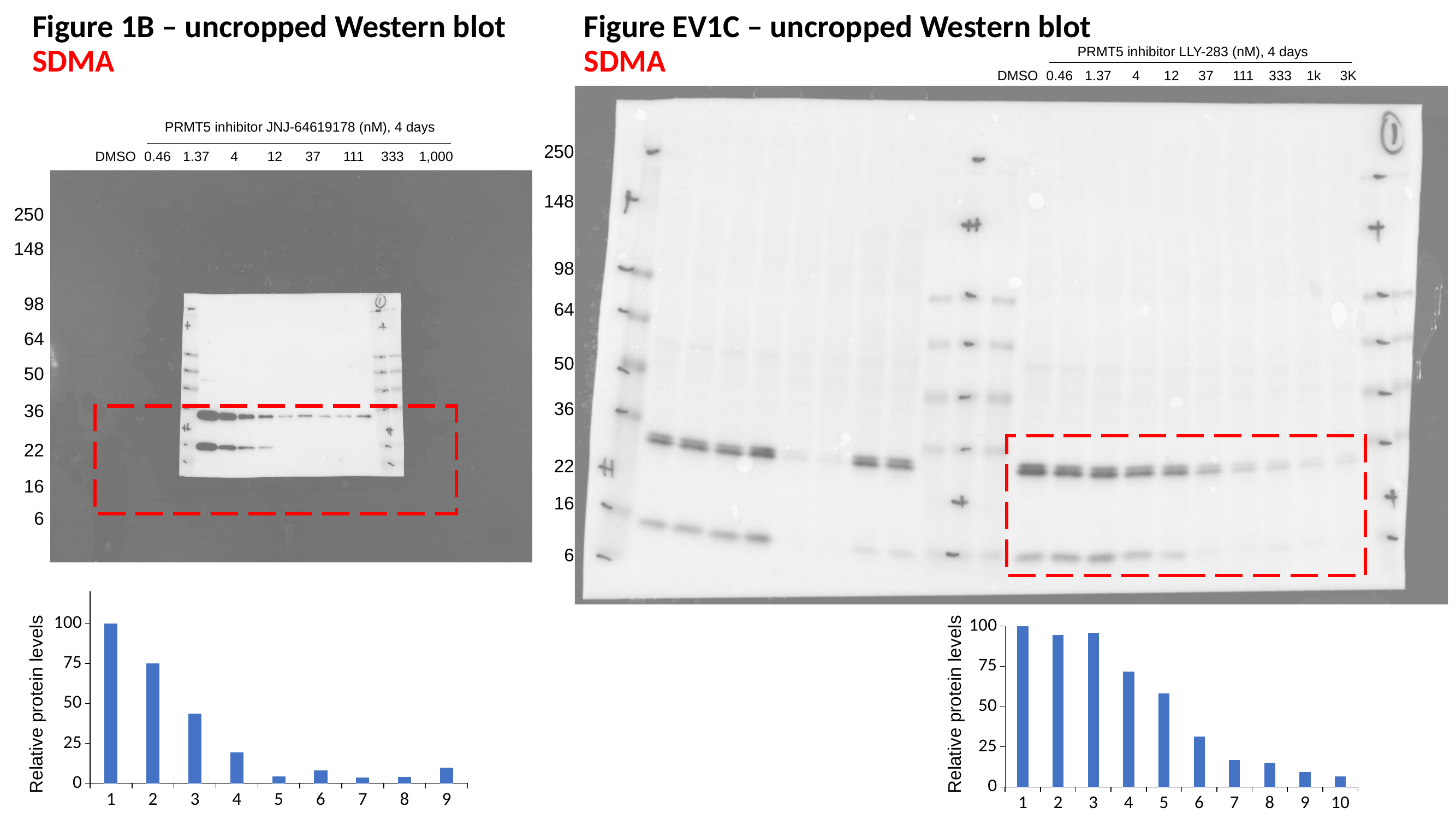

# Figure 1B – uncropped Western blot SDMA
Figure EV1C – uncropped Western blot
SDMA
PRMT5 inhibitor LLY-283 (nM), 4 days
DMSO
0.46
1.37
4
12
37
111
333
1k 3K
PRMT5 inhibitor JNJ-64619178 (nM), 4 days
DMSO
0.46
1.37
4
12
37
111
333
1,000
250
148
250
148
98
98
64
64
50
50
36
36
22
22
16
16
6
### Chart
| Category | |
|---|---|
| 1 | 100.0 |
| 2 | 74.83471739133395 |
| 3 | 43.69434223916811 |
| 4 | 19.334823206562533 |
| 5 | 4.402454190540188 |
| 6 | 8.192985572017832 |
| 7 | 3.748037108746003 |
| 8 | 3.86896085609343 |
| 9 | 9.918631383359608 |6
### Chart
| Category | |
|---|---|
| 1 | 100.0 |
| 2 | 94.4113689463666 |
| 3 | 95.7464669082543 |
| 4 | 71.66356655943228 |
| 5 | 58.204609420350415 |
| 6 | 31.531625029098397 |
| 7 | 16.659098063363725 |
| 8 | 15.117292118838089 |
| 9 | 9.328158056012205 |
| 10 | 6.499762765012311 |Relative protein levels
Relative protein levels

## Slide 3
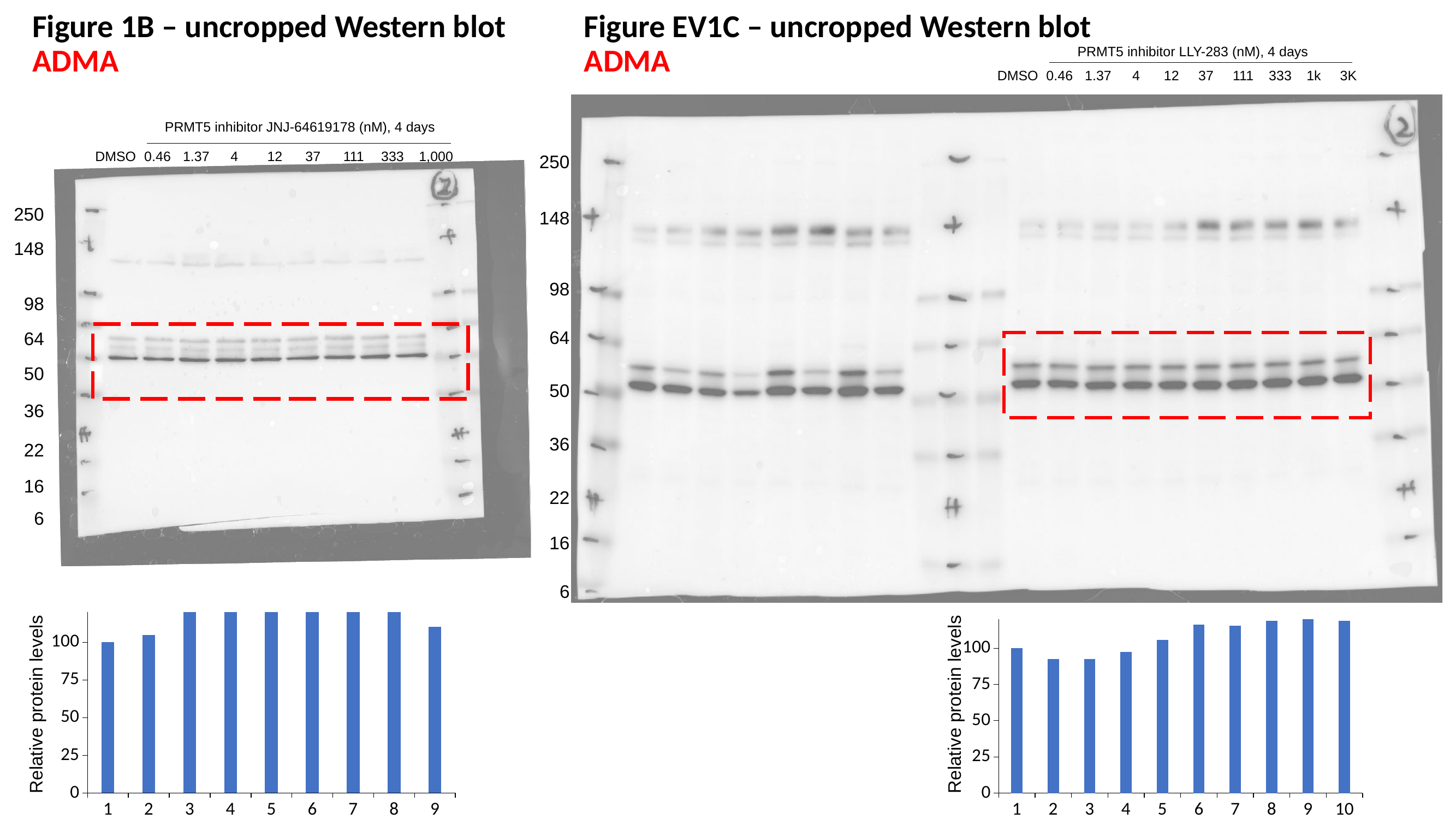

# Figure 1B – uncropped Western blot ADMA
Figure EV1C – uncropped Western blot
ADMA
PRMT5 inhibitor LLY-283 (nM), 4 days
DMSO
0.46
1.37
4
12
37
111
333
1k 3K
PRMT5 inhibitor JNJ-64619178 (nM), 4 days
DMSO
0.46
1.37
4
12
37
111
333
1,000
250
250
148
148
98
98
64
64
50
50
36
36
22
16
22
6
16
### Chart
| Category | |
|---|---|
| 1 | 100.0 |
| 2 | 104.83818939406551 |
| 3 | 124.20740661900776 |
| 4 | 125.8468517108248 |
| 5 | 122.81633295884092 |
| 6 | 121.1680276583395 |
| 7 | 128.1529508848402 |
| 8 | 121.32534512149735 |
| 9 | 110.35864968469289 |
### Chart
| Category | |
|---|---|
| 1 | 100.0 |
| 2 | 92.45867353856421 |
| 3 | 92.40295313718437 |
| 4 | 97.49807487763815 |
| 5 | 105.58022767846167 |
| 6 | 116.25426221520352 |
| 7 | 115.72126811282612 |
| 8 | 118.99161608303706 |
| 9 | 121.30186406203462 |
| 10 | 118.79469756517015 |6
Relative protein levels
Relative protein levels

## Slide 4
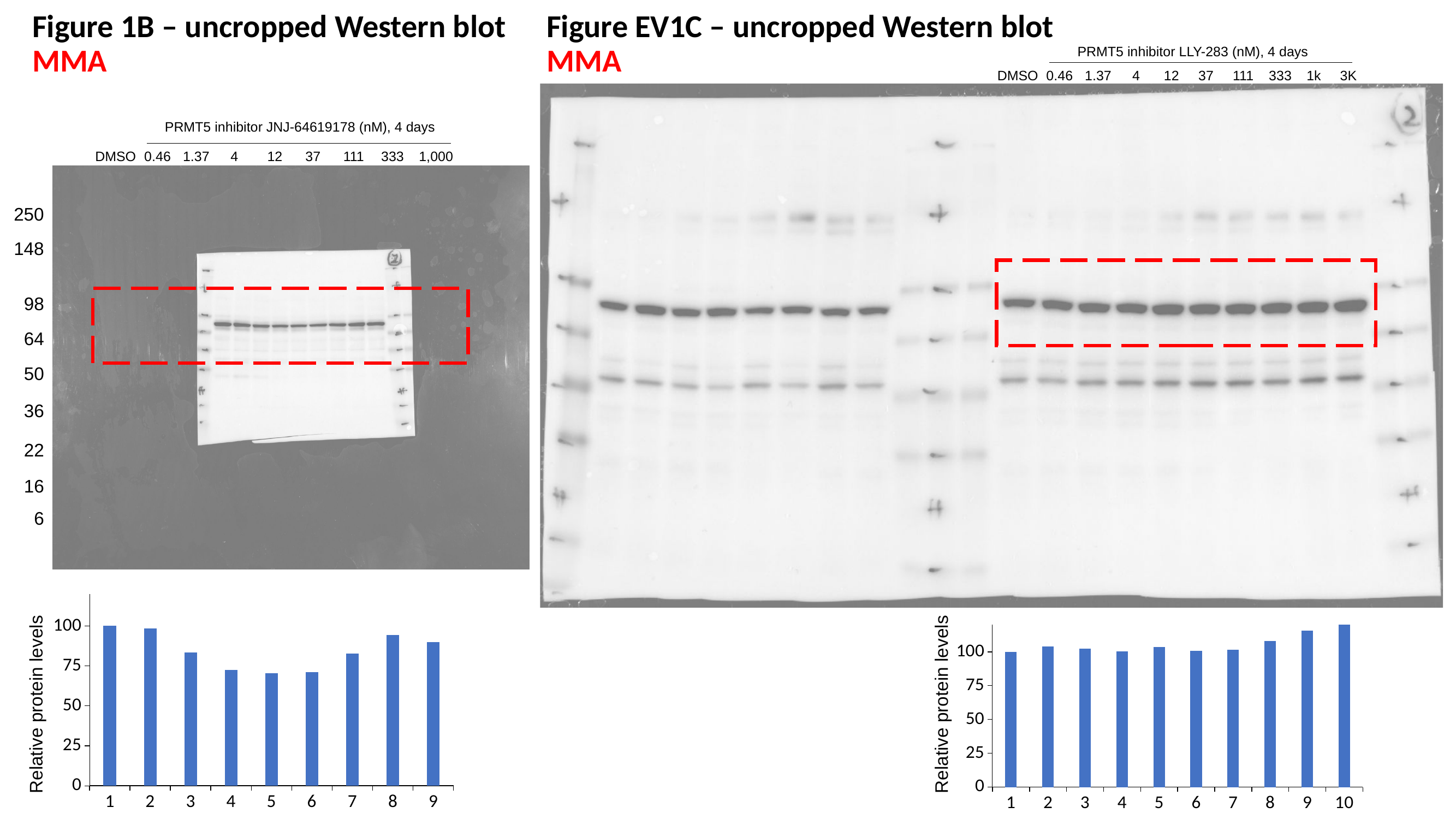

# Figure 1B – uncropped Western blot MMA
Figure EV1C – uncropped Western blot
MMA
PRMT5 inhibitor LLY-283 (nM), 4 days
DMSO
0.46
1.37
4
12
37
111
333
1k 3K
PRMT5 inhibitor JNJ-64619178 (nM), 4 days
DMSO
0.46
1.37
4
12
37
111
333
1,000
250
148
98
64
50
36
22
16
6
### Chart
| Category | |
|---|---|
| 1 | 100.0 |
| 2 | 98.4691026145995 |
| 3 | 83.29753323925289 |
| 4 | 72.59337048681289 |
| 5 | 70.24532172579232 |
| 6 | 70.92933812514094 |
| 7 | 82.55963192099655 |
| 8 | 94.35237739229943 |
| 9 | 90.04320662091638 |
### Chart
| Category | |
|---|---|
| 1 | 100.0 |
| 2 | 104.07090202710141 |
| 3 | 102.23066048791179 |
| 4 | 100.32574440791748 |
| 5 | 103.56276750843504 |
| 6 | 100.6404938398336 |
| 7 | 101.6836482692582 |
| 8 | 107.95923758598491 |
| 9 | 115.65815330377744 |
| 10 | 126.29341479994312 |Relative protein levels
Relative protein levels

## Slide 5
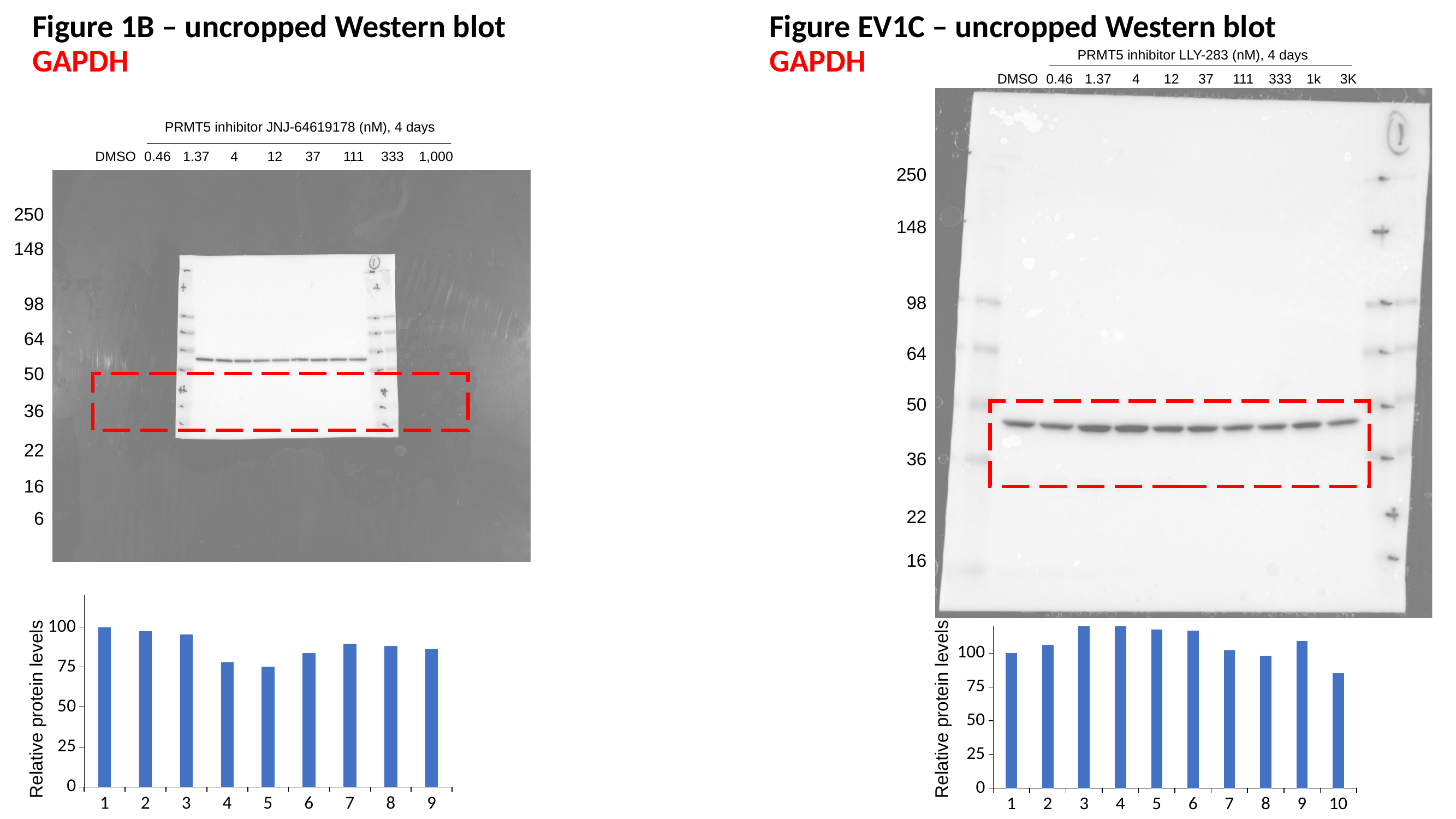

# Figure 1B – uncropped Western blot GAPDH
Figure EV1C – uncropped Western blot
GAPDH
PRMT5 inhibitor LLY-283 (nM), 4 days
DMSO
0.46
1.37
4
12
37
111
333
1k 3K
PRMT5 inhibitor JNJ-64619178 (nM), 4 days
DMSO
0.46
1.37
4
12
37
111
333
1,000
250
250
148
148
98
98
64
64
50
50
36
22
36
16
22
6
### Chart
| Category | |
|---|---|
| 1 | 100.0 |
| 2 | 97.58049578105054 |
| 3 | 95.35403153830093 |
| 4 | 78.07655980292961 |
| 5 | 75.09296372690055 |
| 6 | 83.68616033155061 |
| 7 | 89.70187059339086 |
| 8 | 88.19709793790385 |
| 9 | 86.26573454383875 |16
### Chart
| Category | |
|---|---|
| 1 | 100.0 |
| 2 | 106.09287584468174 |
| 3 | 137.58691919916734 |
| 4 | 142.7257999050029 |
| 5 | 117.44488670800013 |
| 6 | 116.74459371474902 |
| 7 | 102.3575130186802 |
| 8 | 97.95307159244666 |
| 9 | 108.97874592091003 |
| 10 | 85.02693280165845 |Relative protein levels
Relative protein levels

## Slide 6
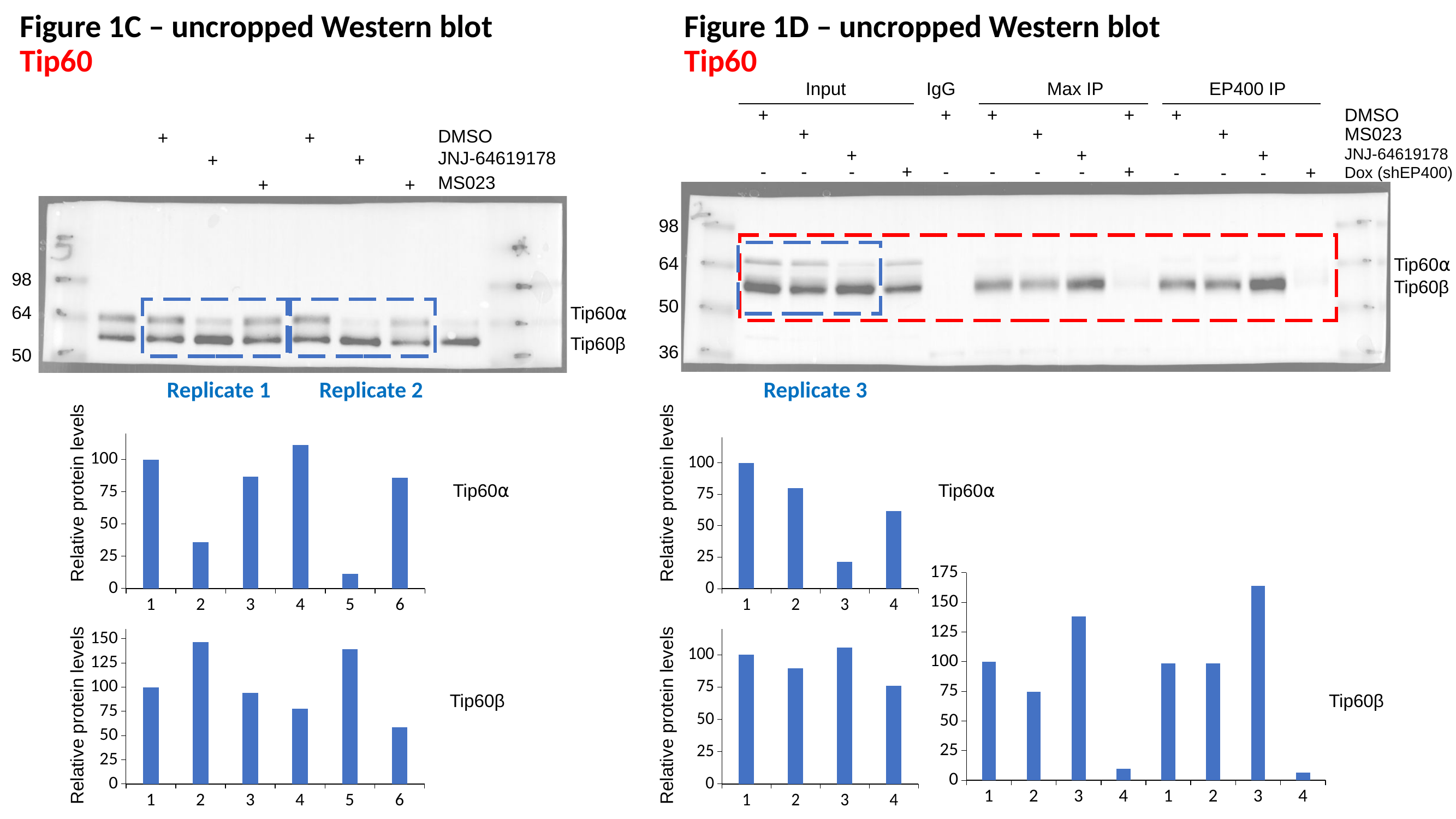

# Figure 1C – uncropped Western blot Tip60
Figure 1D – uncropped Western blot
Tip60
Input
IgG
Max IP
EP400 IP
 +
 +
 +
 +
 +
DMSO
 +
 +
 +
MS023
 +
 +
 +
JNJ-64619178
 -
 -
 -
 +
 -
 -
 -
 -
 +
 -
 -
 -
 +
Dox (shEP400)
 +
 +
DMSO
 +
JNJ-64619178
MS023
 +
 +
 +
98
64
Tip60⍺
Tip60β
50
98
64
Tip60⍺
Tip60β
50
36
Replicate 1
Replicate 2
Replicate 3
### Chart
| Category | |
|---|---|
| 1 | 100.0 |
| 2 | 35.79688193540708 |
| 3 | 86.52096083102417 |
| 4 | 111.08391547775358 |
| 5 | 11.369401832625405 |
| 6 | 85.65505883815955 |
### Chart
| Category | |
|---|---|
| 1 | 100.0 |
| 2 | 80.0622809165321 |
| 3 | 21.25455406925749 |
| 4 | 61.51809540339252 |Tip60⍺
Tip60⍺
Relative protein levels
Relative protein levels
### Chart
| Category | |
|---|---|
| 1 | 100.0 |
| 2 | 74.43859443074805 |
| 3 | 138.00573728397163 |
| 4 | 9.580967674802237 |
| 1 | 98.66204071255927 |
| 2 | 98.38115992790117 |
| 3 | 164.13866992889012 |
| 4 | 6.73362037172155 |
### Chart
| Category | |
|---|---|
| 1 | 100.0 |
| 2 | 146.5538765806235 |
| 3 | 93.97615485176364 |
| 4 | 77.87384090291891 |
| 5 | 139.39800357439637 |
| 6 | 58.45485022075177 |
### Chart
| Category | |
|---|---|
| 1 | 100.0 |
| 2 | 89.54404579857353 |
| 3 | 105.7890963277052 |
| 4 | 75.9108273400297 |Tip60β
Tip60β
Relative protein levels
Relative protein levels

## Slide 7
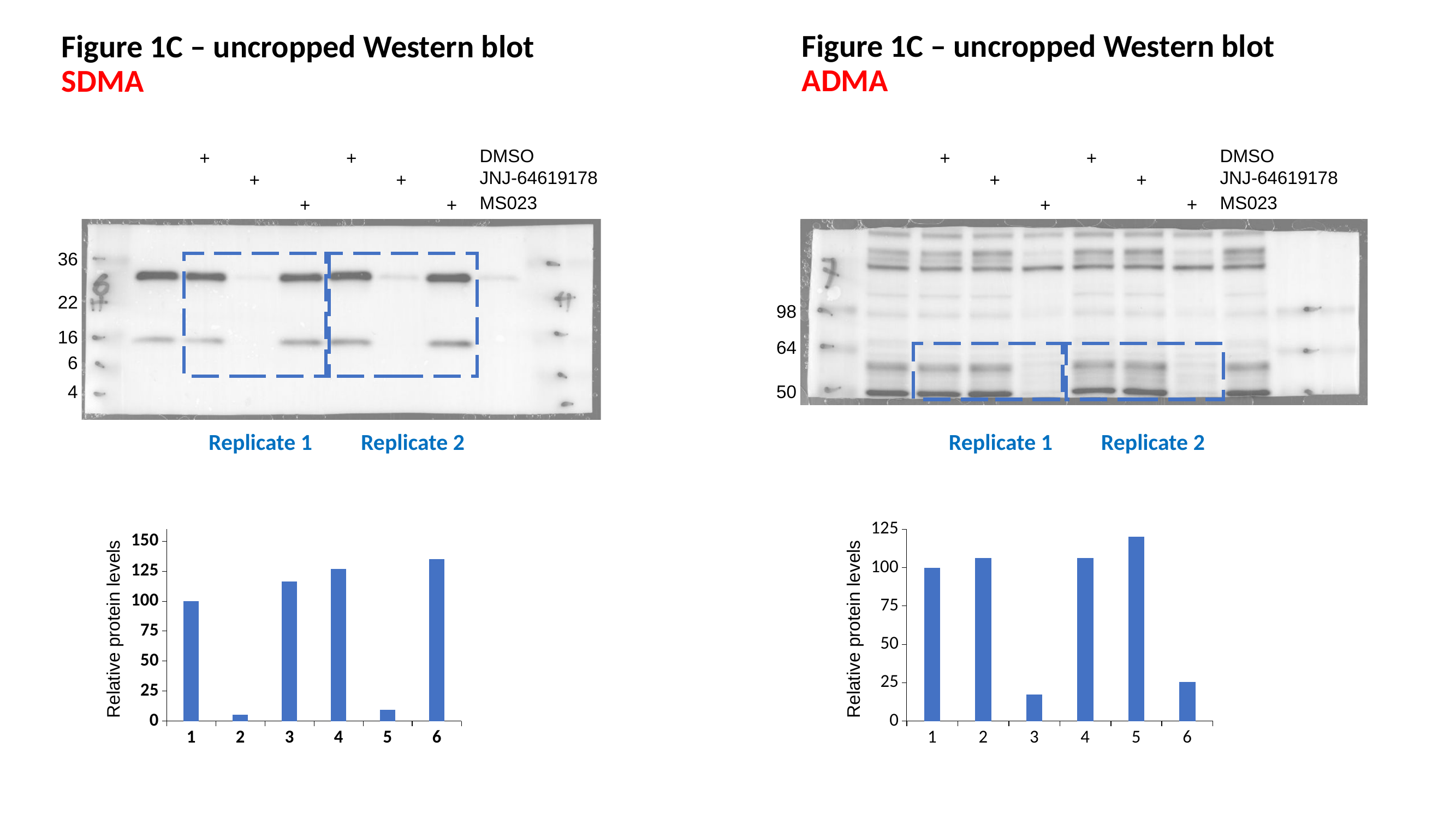

Figure 1C – uncropped Western blot
ADMA
# Figure 1C – uncropped Western blot SDMA
 +
 +
DMSO
 +
JNJ-64619178
MS023
 +
 +
 +
 +
 +
DMSO
 +
JNJ-64619178
MS023
 +
 +
 +
36
22
98
16
64
6
50
4
Replicate 1
Replicate 2
Replicate 1
Replicate 2
### Chart
| Category | |
|---|---|
| 1 | 100.0 |
| 2 | 5.238035667777645 |
| 3 | 116.21341505557625 |
| 4 | 126.88103113079524 |
| 5 | 9.528210552998784 |
| 6 | 135.16977449365186 |
### Chart
| Category | |
|---|---|
| 1 | 100.0 |
| 2 | 106.3234889007191 |
| 3 | 17.211448272365452 |
| 4 | 106.40594130521121 |
| 5 | 119.98286730718854 |
| 6 | 25.456925325964797 |Relative protein levels
Relative protein levels

## Slide 8
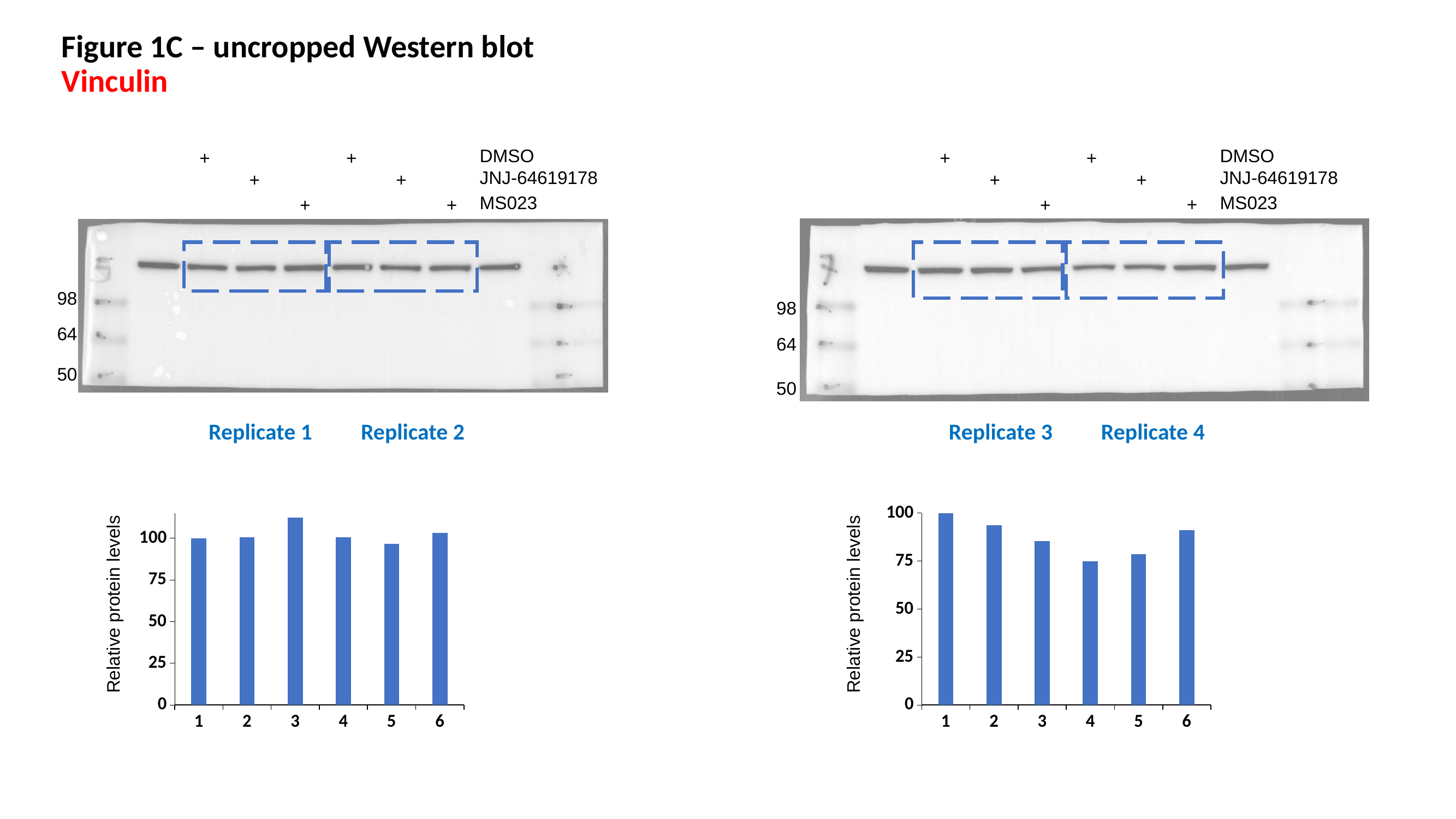

# Figure 1C – uncropped Western blot Vinculin
 +
 +
DMSO
 +
JNJ-64619178
MS023
 +
 +
 +
 +
 +
DMSO
 +
JNJ-64619178
MS023
 +
 +
 +
98
98
64
64
50
50
Replicate 1
Replicate 2
Replicate 3
Replicate 4
### Chart
| Category | |
|---|---|
| 1 | 100.0 |
| 2 | 100.50881949916896 |
| 3 | 112.23555684426773 |
| 4 | 100.53621017808683 |
| 5 | 96.61950944982583 |
| 6 | 103.01249224568467 |
### Chart
| Category | |
|---|---|
| 1 | 100.0 |
| 2 | 93.74046911434584 |
| 3 | 85.5029422557881 |
| 4 | 74.95071507444223 |
| 5 | 78.66854489429484 |
| 6 | 91.15371994921965 |Relative protein levels
Relative protein levels

## Slide 9
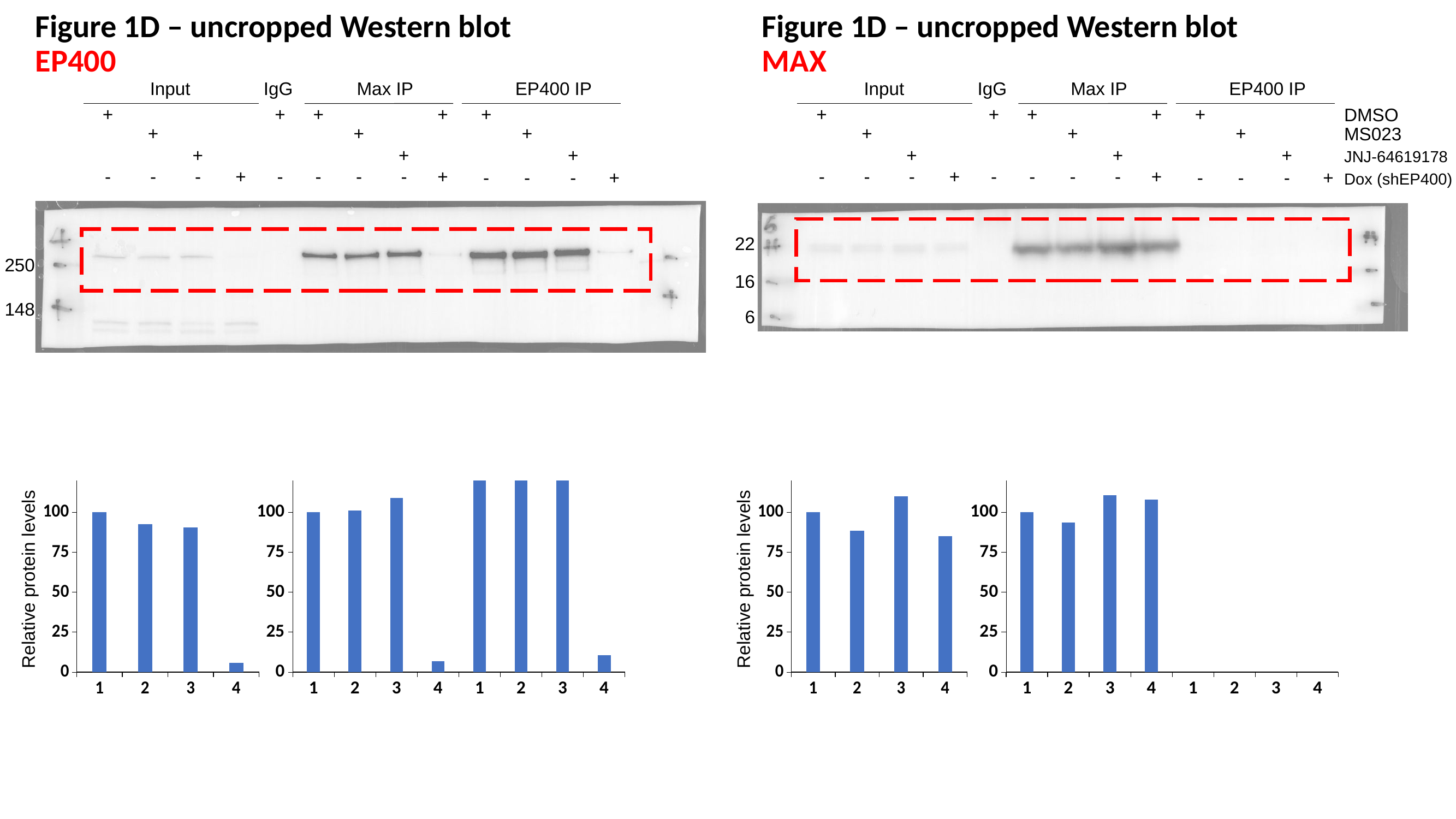

# Figure 1D – uncropped Western blot EP400
Figure 1D – uncropped Western blot
MAX
Input
IgG
Max IP
EP400 IP
 +
 +
 +
 +
 +
 +
 +
 +
 +
 +
 +
 -
 -
 -
 +
 -
 -
 -
 -
 +
 -
 -
 -
 +
Input
IgG
Max IP
EP400 IP
 +
 +
 +
 +
 +
 +
 +
 +
 +
 +
 +
 -
 -
 -
 +
 -
 -
 -
 -
 +
 -
 -
 -
 +
DMSO
MS023
JNJ-64619178
Dox (shEP400)
22
250
16
148
6
### Chart
| Category | |
|---|---|
| 1 | 100.0 |
| 2 | 92.64954943805806 |
| 3 | 90.65410547641312 |
| 4 | 5.783854023431054 |
### Chart
| Category | |
|---|---|
| 1 | 100.0 |
| 2 | 101.1848721968858 |
| 3 | 109.08679422525675 |
| 4 | 6.914248541535054 |
| 1 | 153.21078526180432 |
| 2 | 156.98165707394162 |
| 3 | 151.2242731711284 |
| 4 | 10.585487191517338 |
### Chart
| Category | |
|---|---|
| 1 | 100.0 |
| 2 | 88.59181728598452 |
| 3 | 109.8601004319249 |
| 4 | 84.91264905764574 |
### Chart
| Category | |
|---|---|
| 1 | 100.0 |
| 2 | 93.66013019014305 |
| 3 | 110.7740838729455 |
| 4 | 107.76436201406064 |
| 1 | None |
| 2 | None |
| 3 | None |
| 4 | None |Relative protein levels
Relative protein levels

## Slide 10
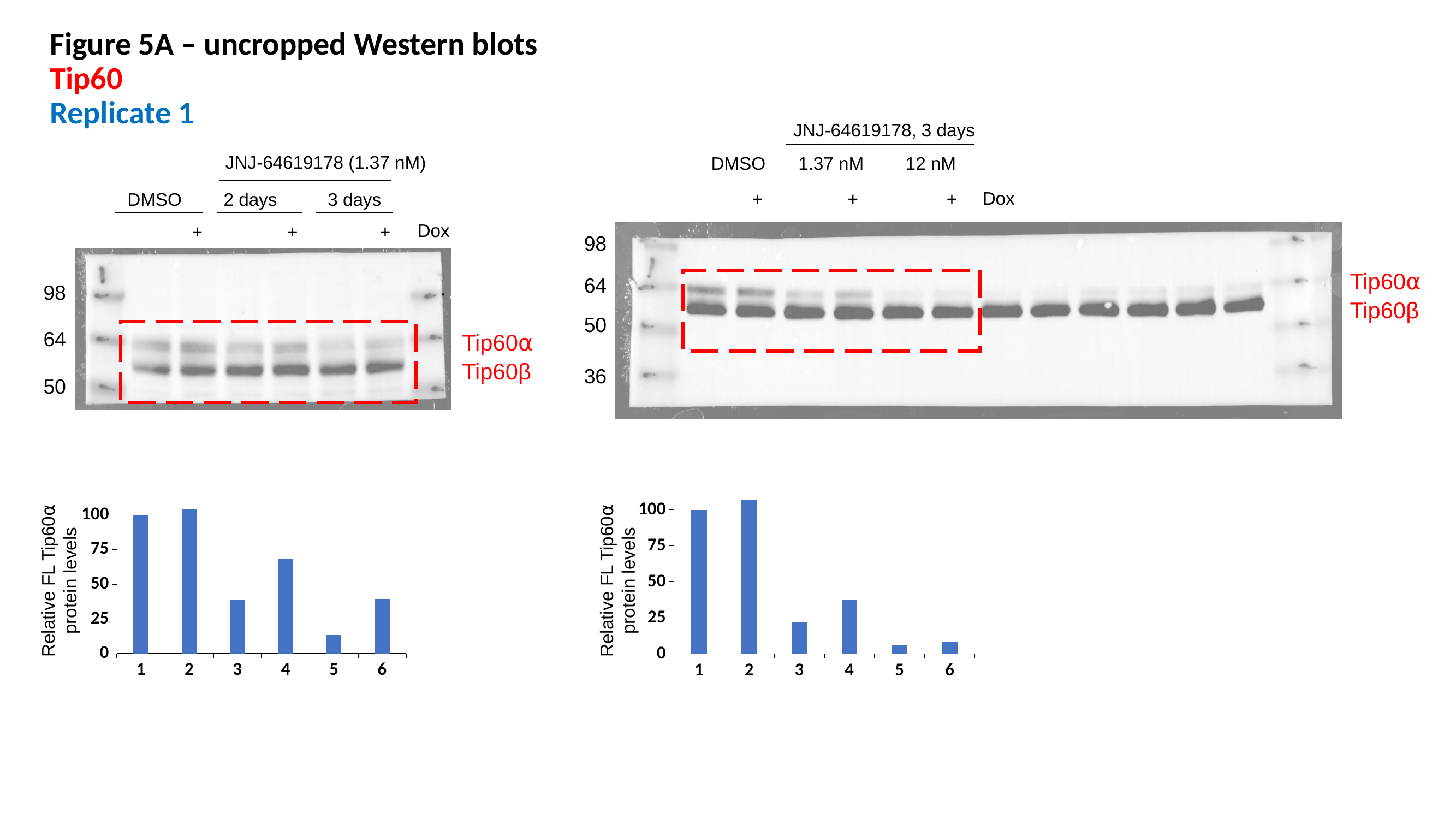

# Figure 5A – uncropped Western blots Tip60 Replicate 1
JNJ-64619178, 3 days
DMSO
1.37 nM
12 nM
 +
 +
 +
Dox
JNJ-64619178 (1.37 nM)
DMSO
2 days
3 days
 +
 +
 +
Dox
98
Tip60⍺
64
98
Tip60β
50
64
Tip60⍺
Tip60β
36
50
### Chart
| Category | |
|---|---|
### Chart
| Category | |
|---|---|Relative FL Tip60⍺ protein levels
Relative FL Tip60⍺ protein levels

## Slide 11
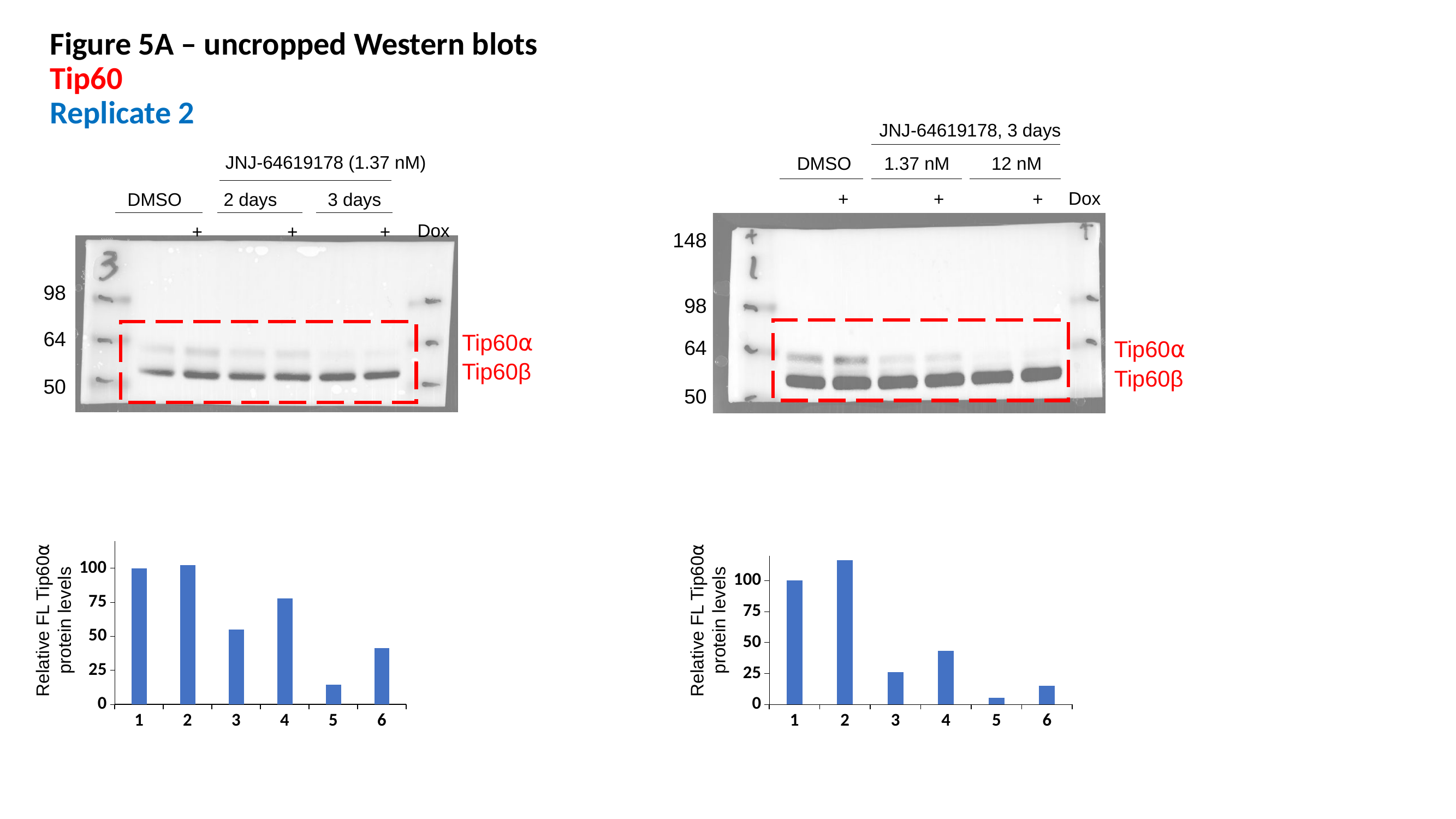

# Figure 5A – uncropped Western blots Tip60 Replicate 2
JNJ-64619178, 3 days
DMSO
1.37 nM
12 nM
 +
 +
 +
Dox
JNJ-64619178 (1.37 nM)
DMSO
2 days
3 days
 +
 +
 +
Dox
148
98
98
64
Tip60⍺
64
Tip60⍺
Tip60β
Tip60β
50
50
### Chart
| Category | |
|---|---|
### Chart
| Category | |
|---|---|Relative FL Tip60⍺ protein levels
Relative FL Tip60⍺ protein levels

## Slide 12
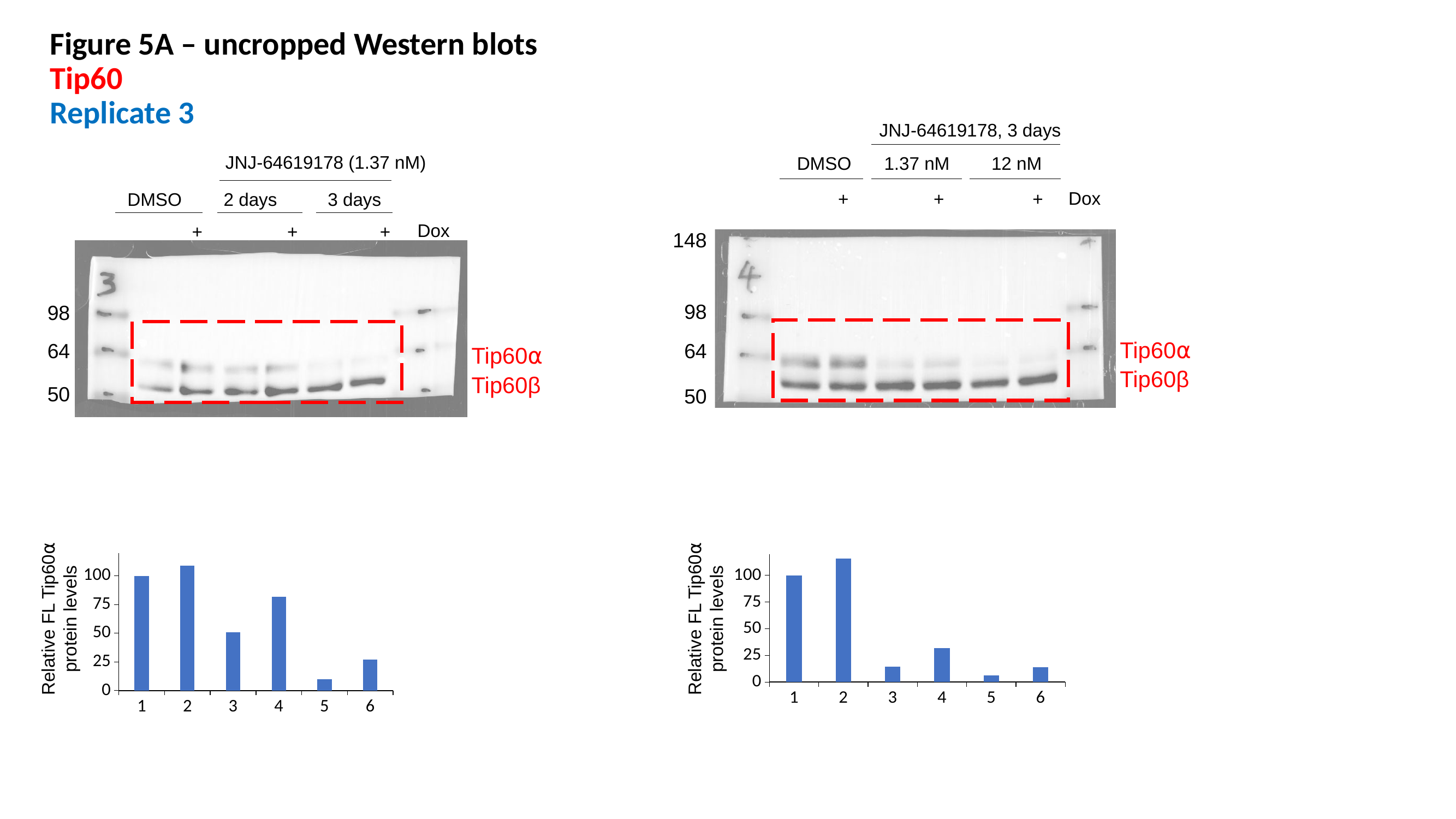

# Figure 5A – uncropped Western blots Tip60 Replicate 3
JNJ-64619178, 3 days
DMSO
1.37 nM
12 nM
 +
 +
 +
Dox
JNJ-64619178 (1.37 nM)
DMSO
2 days
3 days
 +
 +
 +
Dox
148
98
98
Tip60⍺
64
64
Tip60⍺
Tip60β
Tip60β
50
50
### Chart
| Category | |
|---|---|
### Chart
| Category | |
|---|---|Relative FL Tip60⍺ protein levels
Relative FL Tip60⍺ protein levels

## Slide 13
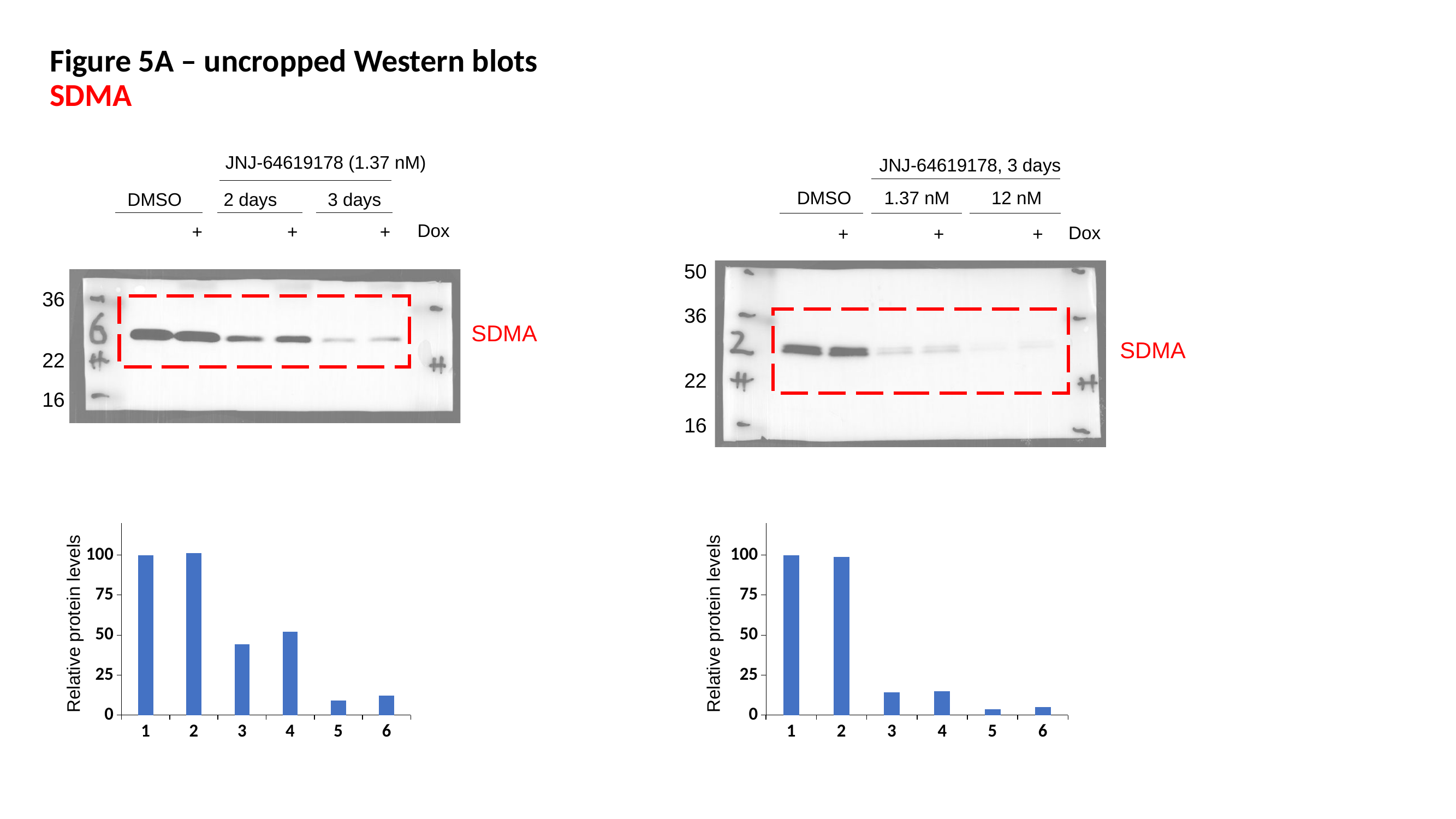

# Figure 5A – uncropped Western blots SDMA
JNJ-64619178 (1.37 nM)
JNJ-64619178, 3 days
DMSO
1.37 nM
12 nM
 +
 +
 +
Dox
DMSO
2 days
3 days
 +
 +
 +
Dox
50
36
36
SDMA
SDMA
22
22
16
16
### Chart
| Category | |
|---|---|
### Chart
| Category | |
|---|---|Relative protein levels
Relative protein levels

## Slide 14
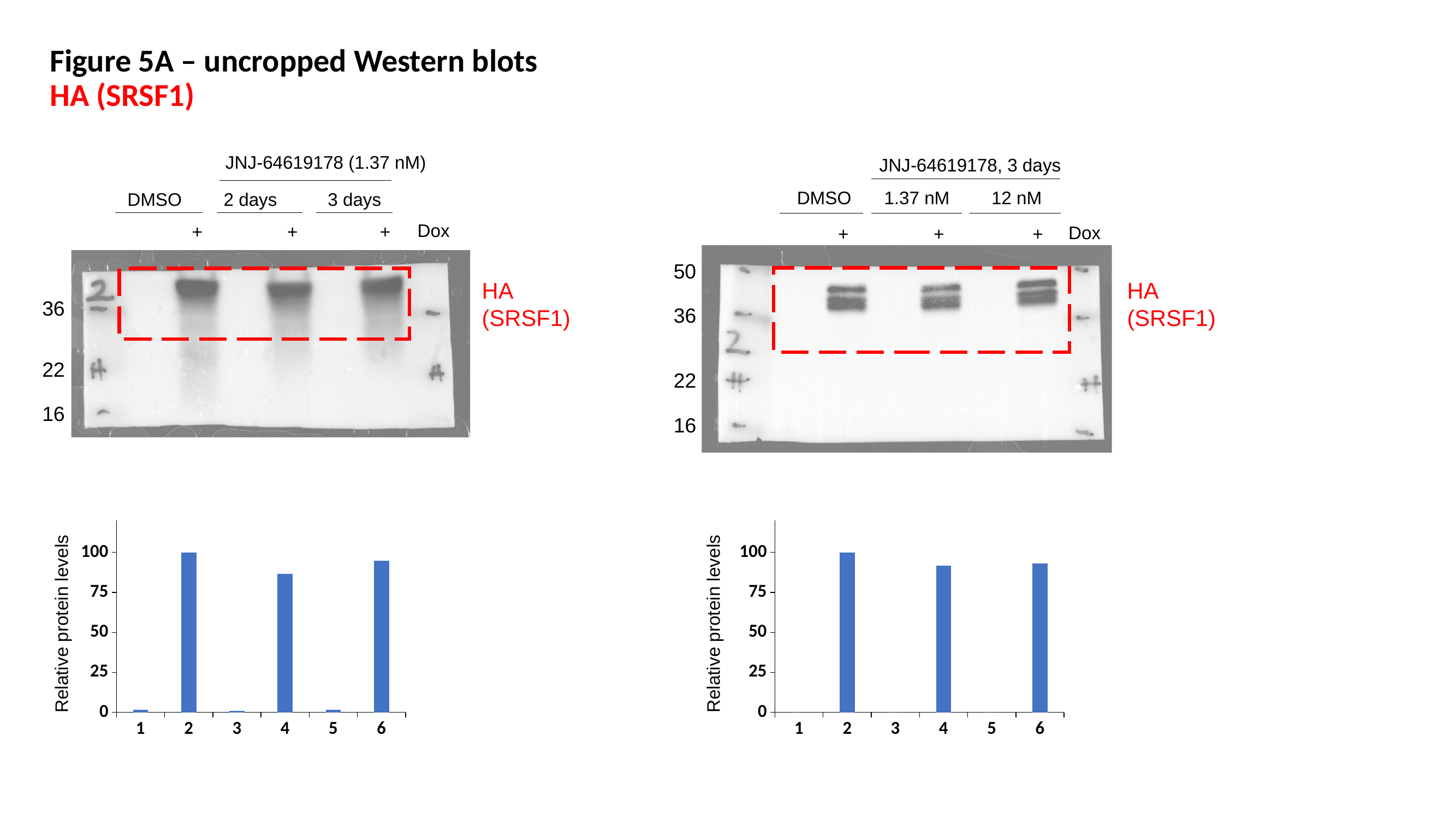

# Figure 5A – uncropped Western blots HA (SRSF1)
JNJ-64619178 (1.37 nM)
JNJ-64619178, 3 days
DMSO
1.37 nM
12 nM
 +
 +
 +
Dox
DMSO
2 days
3 days
 +
 +
 +
Dox
50
HA (SRSF1)
HA (SRSF1)
36
36
22
22
16
16
### Chart
| Category | |
|---|---|
### Chart
| Category | |
|---|---|Relative protein levels
Relative protein levels

## Slide 15
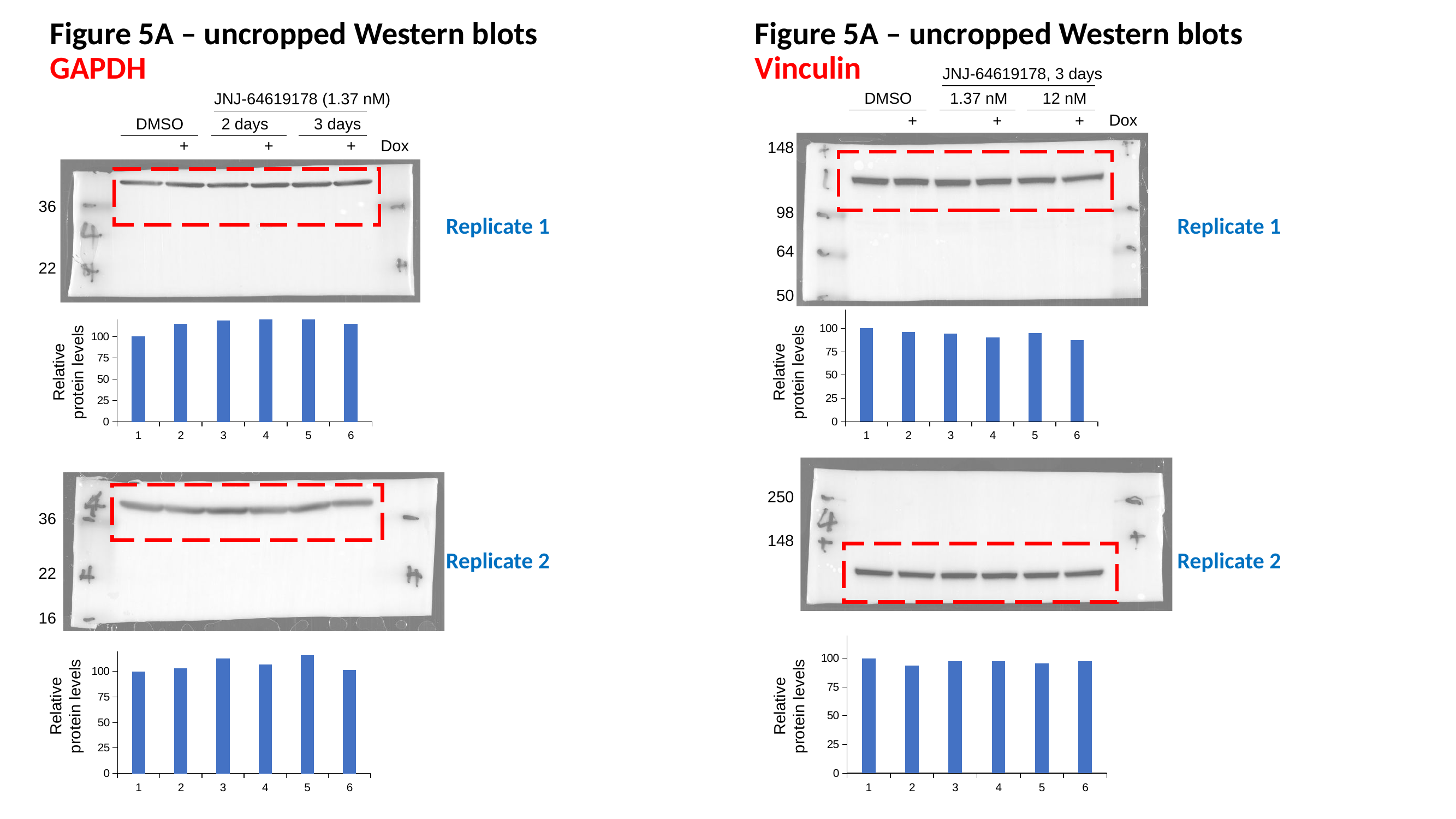

# Figure 5A – uncropped Western blots GAPDH
Figure 5A – uncropped Western blots
Vinculin
JNJ-64619178, 3 days
DMSO
1.37 nM
12 nM
 +
 +
 +
Dox
JNJ-64619178 (1.37 nM)
DMSO
2 days
3 days
 +
 +
 +
Dox
148
36
98
Replicate 1
Replicate 1
64
22
### Chart
| Category | |
|---|---|
### Chart
| Category | |
|---|---|50
Relative protein levels
Relative protein levels
250
36
148
Replicate 2
Replicate 2
22
### Chart
| Category | |
|---|---|
### Chart
| Category | |
|---|---|16
Relative protein levels
Relative protein levels

## Slide 16
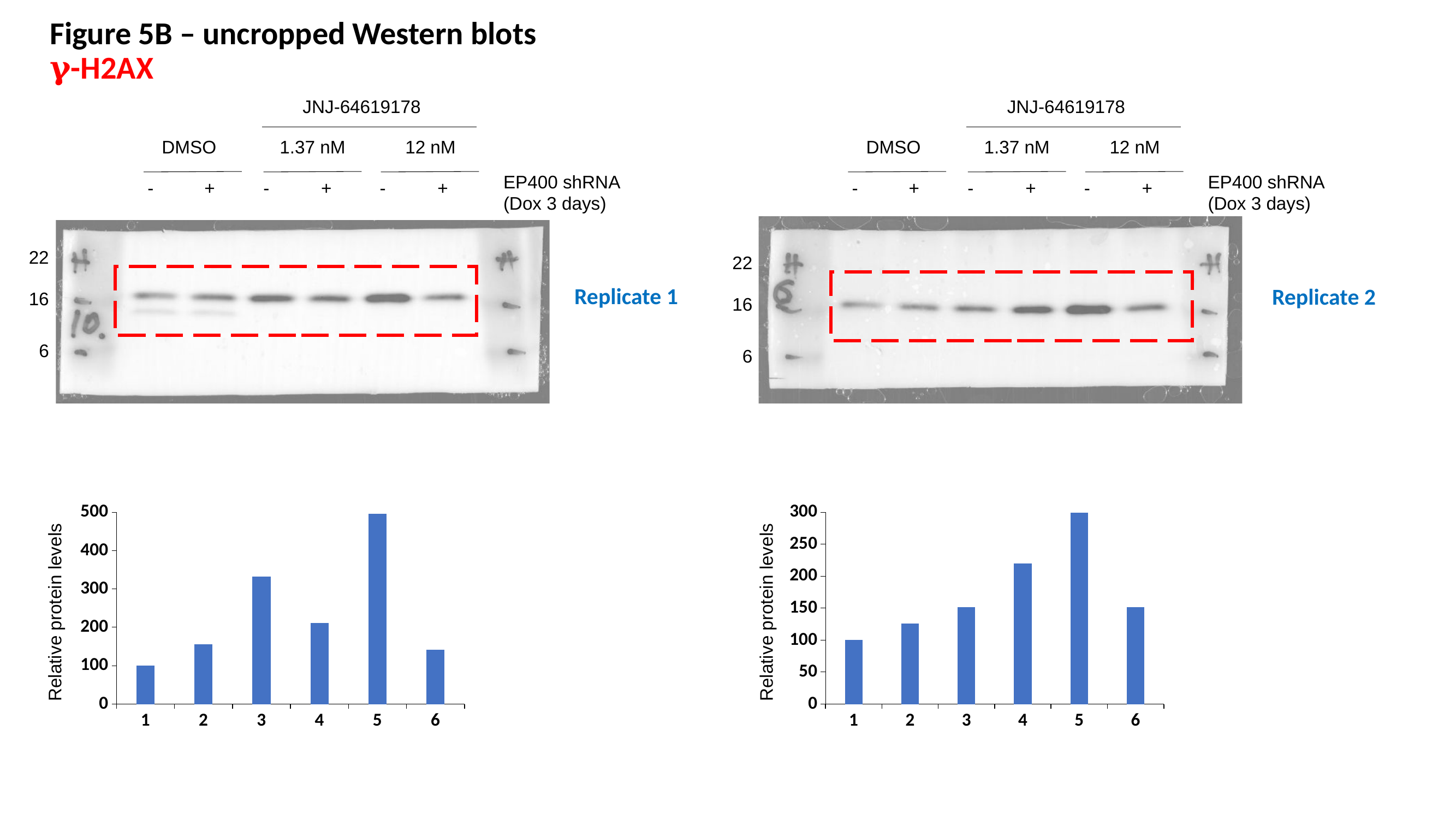

# Figure 5B – uncropped Western blots 𝛄-H2AX
JNJ-64619178
DMSO
1.37 nM
12 nM
EP400 shRNA
(Dox 3 days)
 -
 +
 -
 +
 -
 +
JNJ-64619178
DMSO
1.37 nM
12 nM
EP400 shRNA
(Dox 3 days)
 -
 +
 -
 +
 -
 +
22
22
Replicate 1
Replicate 2
16
16
6
6
### Chart
| Category | |
|---|---|
| 1 | 100.0 |
| 2 | 156.08937034137838 |
| 3 | 332.3324968862188 |
| 4 | 210.888497711419 |
| 5 | 495.770728335602 |
| 6 | 141.65027031091537 |
### Chart
| Category | |
|---|---|
| 1 | 100.0 |
| 2 | 126.00123906524918 |
| 3 | 151.82167372933858 |
| 4 | 219.94873243625008 |
| 5 | 299.03224047778355 |
| 6 | 151.41355537382597 |Relative protein levels
Relative protein levels

## Slide 17
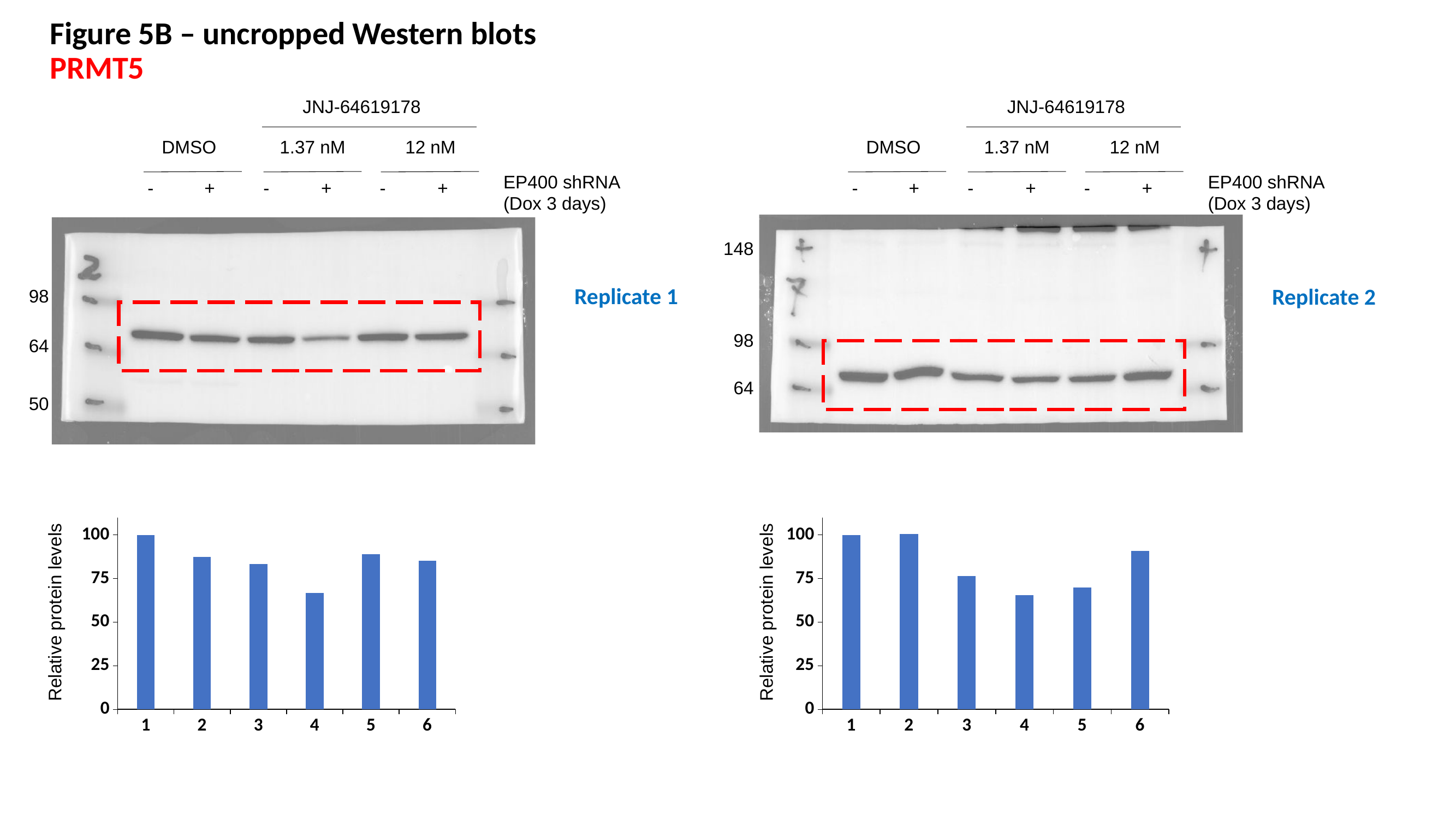

# Figure 5B – uncropped Western blots PRMT5
JNJ-64619178
DMSO
1.37 nM
12 nM
EP400 shRNA
(Dox 3 days)
 -
 +
 -
 +
 -
 +
JNJ-64619178
DMSO
1.37 nM
12 nM
EP400 shRNA
(Dox 3 days)
 -
 +
 -
 +
 -
 +
148
Replicate 1
Replicate 2
98
98
64
64
50
### Chart
| Category | |
|---|---|
| 1 | 100.0 |
| 2 | 87.34008181033418 |
| 3 | 83.20865450943774 |
| 4 | 66.53773508219146 |
| 5 | 88.82187349987336 |
| 6 | 85.24146201047553 |
### Chart
| Category | |
|---|---|
| 1 | 100.0 |
| 2 | 100.40362183843901 |
| 3 | 76.4754910580791 |
| 4 | 65.29340880317068 |
| 5 | 69.83565538255029 |
| 6 | 90.83393748152659 |Relative protein levels
Relative protein levels

## Slide 18
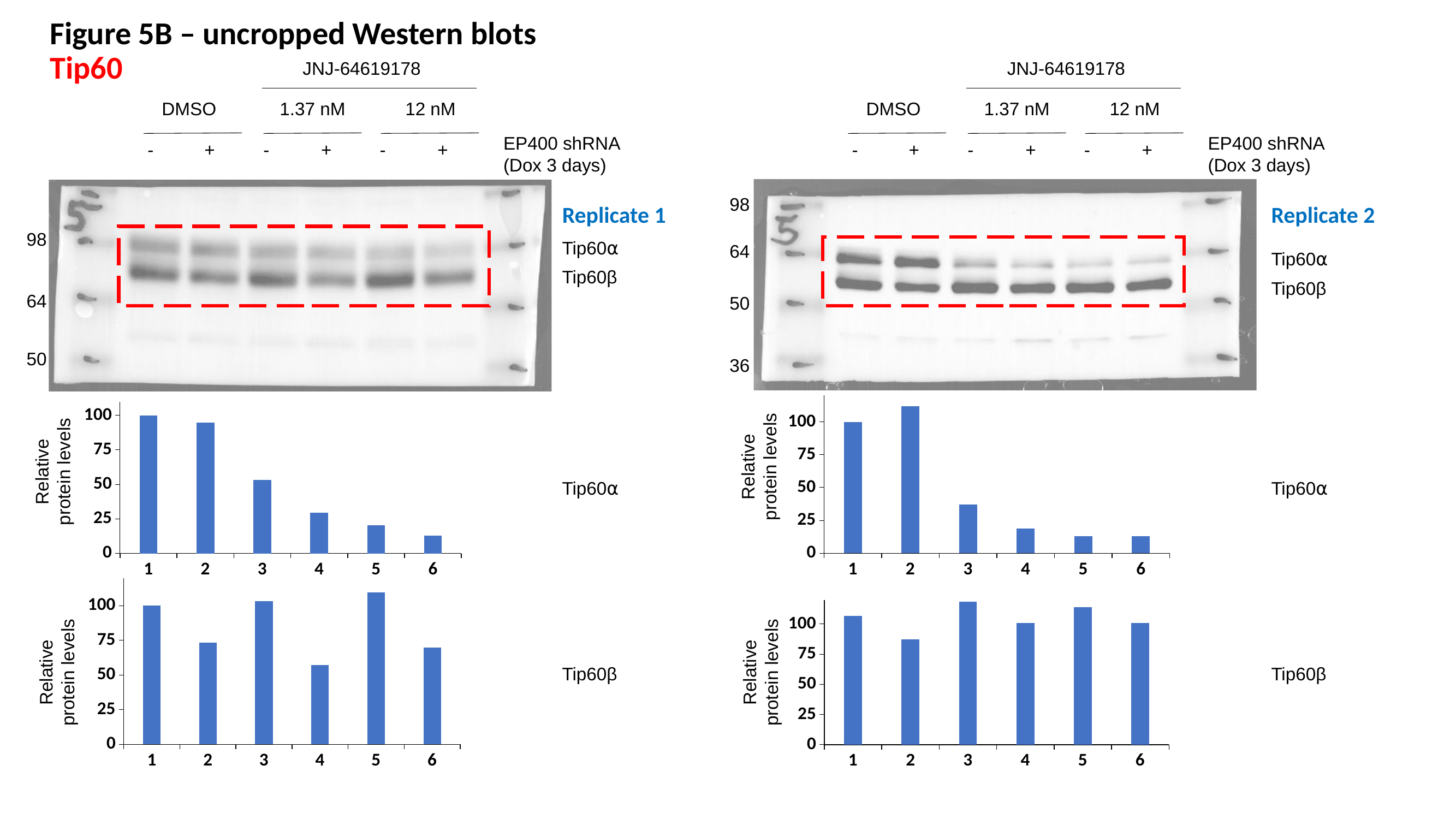

# Figure 5B – uncropped Western blots Tip60
JNJ-64619178
DMSO
1.37 nM
12 nM
EP400 shRNA
(Dox 3 days)
 -
 +
 -
 +
 -
 +
JNJ-64619178
DMSO
1.37 nM
12 nM
EP400 shRNA
(Dox 3 days)
 -
 +
 -
 +
 -
 +
98
Replicate 1
Replicate 2
98
Tip60⍺
64
Tip60⍺
Tip60β
Tip60β
64
50
### Chart
| Category | |
|---|---|
| 1 | 100.0 |
| 2 | 111.68723639180978 |
| 3 | 37.16498447033534 |
| 4 | 18.851088814271947 |
| 5 | 12.906135638436044 |
| 6 | 12.879950972597154 |
### Chart
| Category | |
|---|---|
| 1 | 100.0 |
| 2 | 94.75564903593117 |
| 3 | 53.373238805074465 |
| 4 | 29.584336233267805 |
| 5 | 20.429999738528657 |
| 6 | 12.719337184379178 |50
36
Relative protein levels
Relative protein levels
Tip60⍺
Tip60⍺
### Chart
| Category | |
|---|---|
| 1 | 100.0 |
| 2 | 73.43412991779263 |
| 3 | 103.2228253533413 |
| 4 | 57.16954880826772 |
| 5 | 109.51106012624481 |
| 6 | 69.88135187320952 |
### Chart
| Category | |
|---|---|
| 1 | 106.72681659844278 |
| 2 | 87.08331940563788 |
| 3 | 118.5939024734833 |
| 4 | 100.96101996184932 |
| 5 | 114.07335490698951 |
| 6 | 101.03246859069806 |Relative protein levels
Relative protein levels
Tip60β
Tip60β

## Slide 19
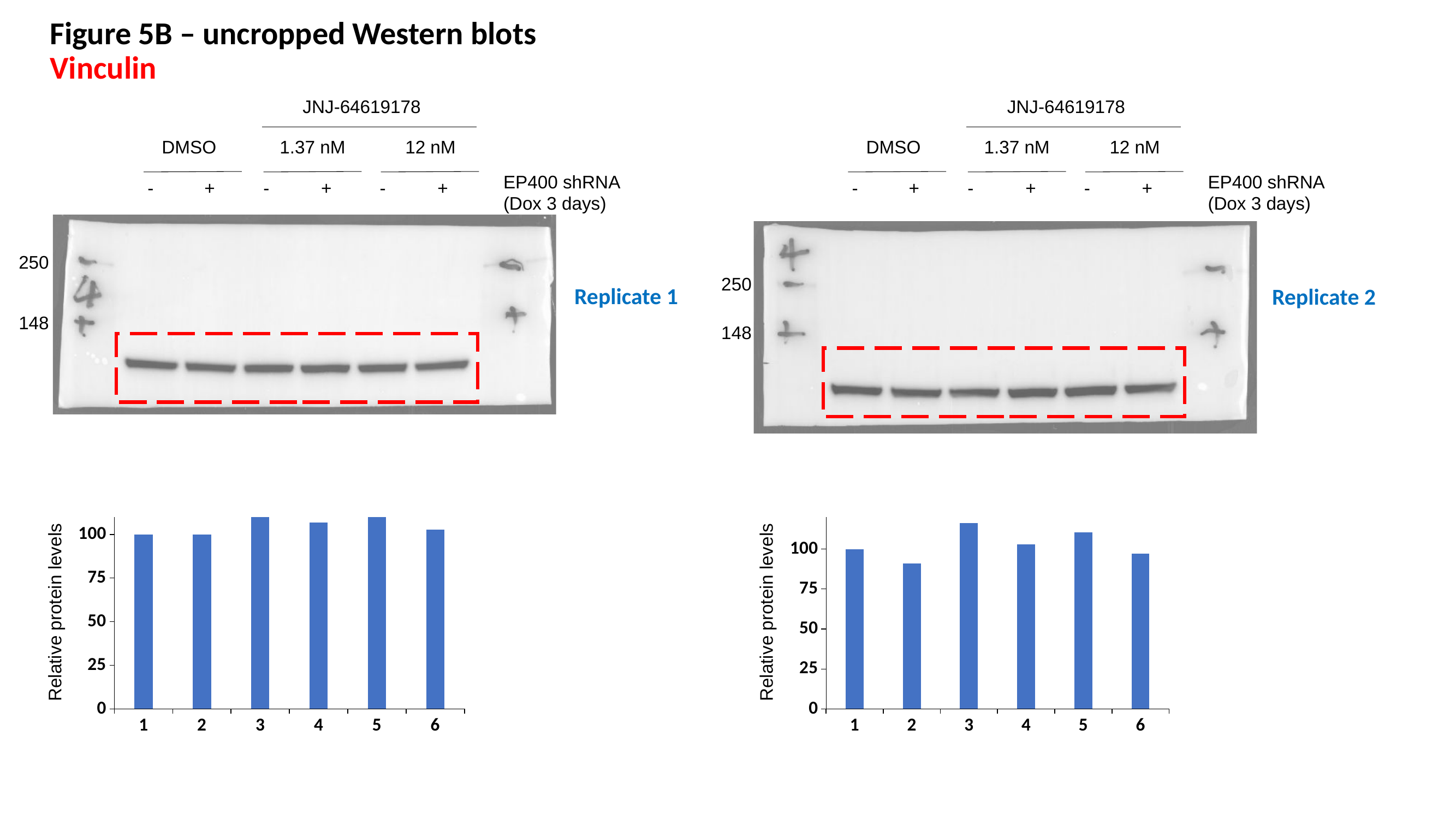

# Figure 5B – uncropped Western blots Vinculin
JNJ-64619178
DMSO
1.37 nM
12 nM
EP400 shRNA
(Dox 3 days)
 -
 +
 -
 +
 -
 +
JNJ-64619178
DMSO
1.37 nM
12 nM
EP400 shRNA
(Dox 3 days)
 -
 +
 -
 +
 -
 +
250
250
Replicate 1
Replicate 2
148
148
### Chart
| Category | |
|---|---|
| 1 | 100.0 |
| 2 | 100.09413411249879 |
| 3 | 121.26978108613437 |
| 4 | 106.80832066927388 |
| 5 | 137.56055924835988 |
| 6 | 102.97536598367381 |
### Chart
| Category | |
|---|---|
| 1 | 100.0 |
| 2 | 91.13590990583887 |
| 3 | 116.41663556321478 |
| 4 | 103.06264896168884 |
| 5 | 110.57470152929119 |
| 6 | 97.04564578399952 |Relative protein levels
Relative protein levels

## Slide 20
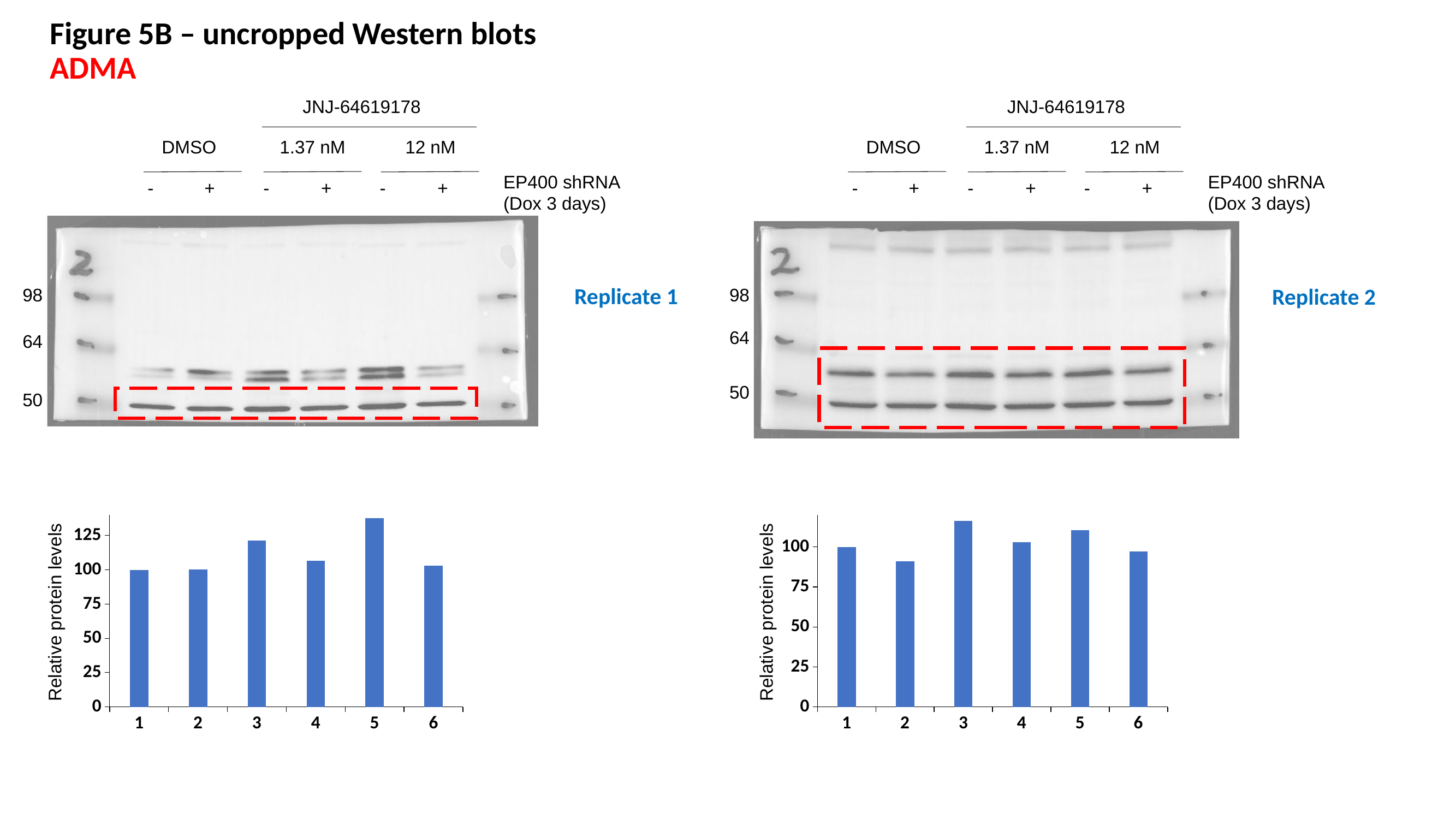

# Figure 5B – uncropped Western blots ADMA
JNJ-64619178
DMSO
1.37 nM
12 nM
EP400 shRNA
(Dox 3 days)
 -
 +
 -
 +
 -
 +
JNJ-64619178
DMSO
1.37 nM
12 nM
EP400 shRNA
(Dox 3 days)
 -
 +
 -
 +
 -
 +
Replicate 1
Replicate 2
98
98
64
64
50
50
### Chart
| Category | |
|---|---|
| 1 | 100.0 |
| 2 | 100.09413411249879 |
| 3 | 121.26978108613437 |
| 4 | 106.80832066927388 |
| 5 | 137.56055924835988 |
| 6 | 102.97536598367381 |
### Chart
| Category | |
|---|---|
| 1 | 100.0 |
| 2 | 91.13590990583887 |
| 3 | 116.41663556321478 |
| 4 | 103.06264896168884 |
| 5 | 110.57470152929119 |
| 6 | 97.04564578399952 |Relative protein levels
Relative protein levels

## Slide 21
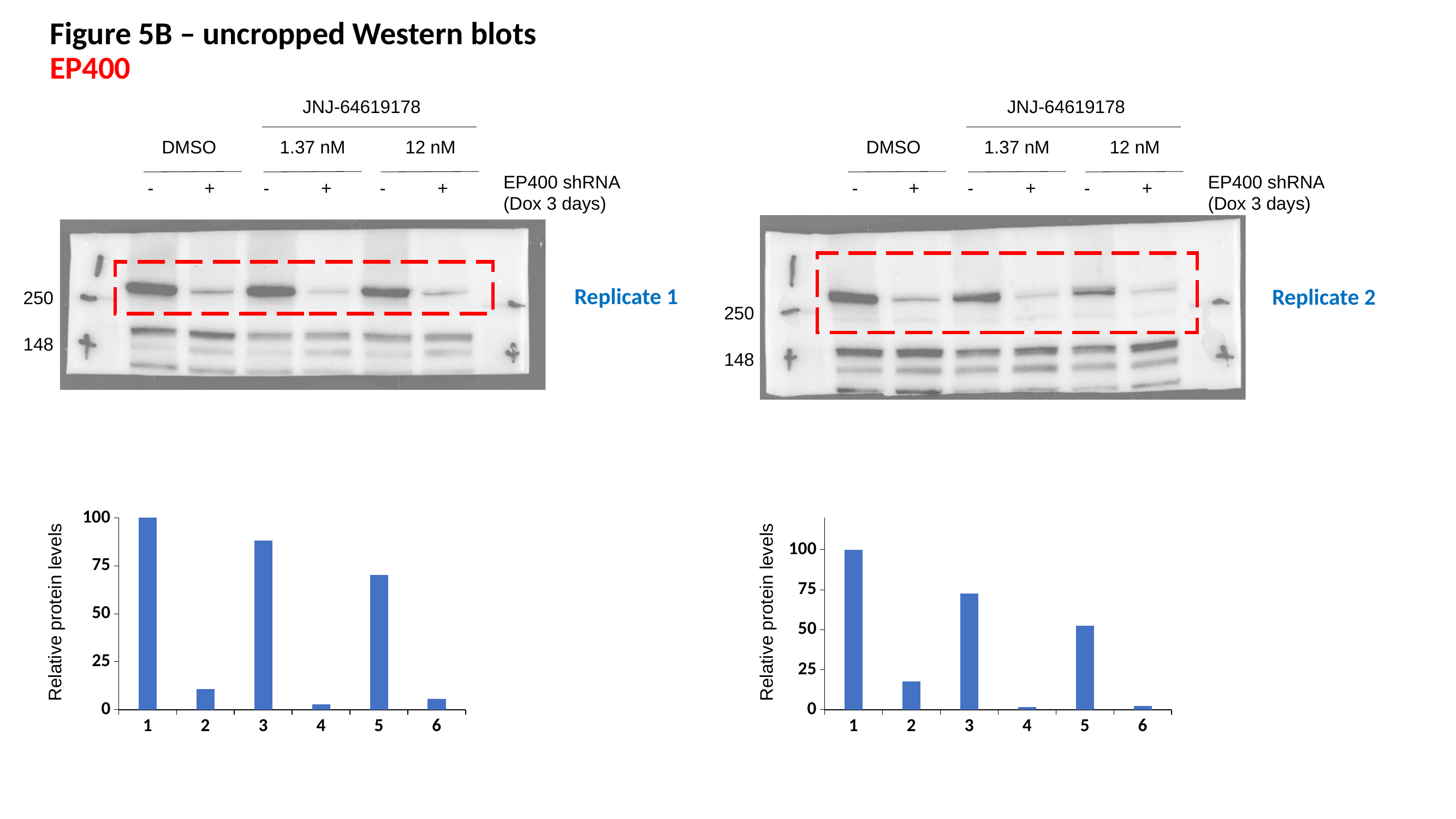

# Figure 5B – uncropped Western blots EP400
JNJ-64619178
DMSO
1.37 nM
12 nM
EP400 shRNA
(Dox 3 days)
 -
 +
 -
 +
 -
 +
JNJ-64619178
DMSO
1.37 nM
12 nM
EP400 shRNA
(Dox 3 days)
 -
 +
 -
 +
 -
 +
Replicate 1
Replicate 2
250
250
148
148
### Chart
| Category | |
|---|---|
| 1 | 100.0 |
| 2 | 10.681429375732288 |
| 3 | 88.16086282149955 |
| 4 | 2.669573093010057 |
| 5 | 70.2881820944262 |
| 6 | 5.6111164499529425 |
### Chart
| Category | |
|---|---|
| 1 | 100.0 |
| 2 | 17.650472263387204 |
| 3 | 72.54978547760801 |
| 4 | 1.6488855629029384 |
| 5 | 52.391967902578585 |
| 6 | 2.169973147616821 |Relative protein levels
Relative protein levels

## Slide 22
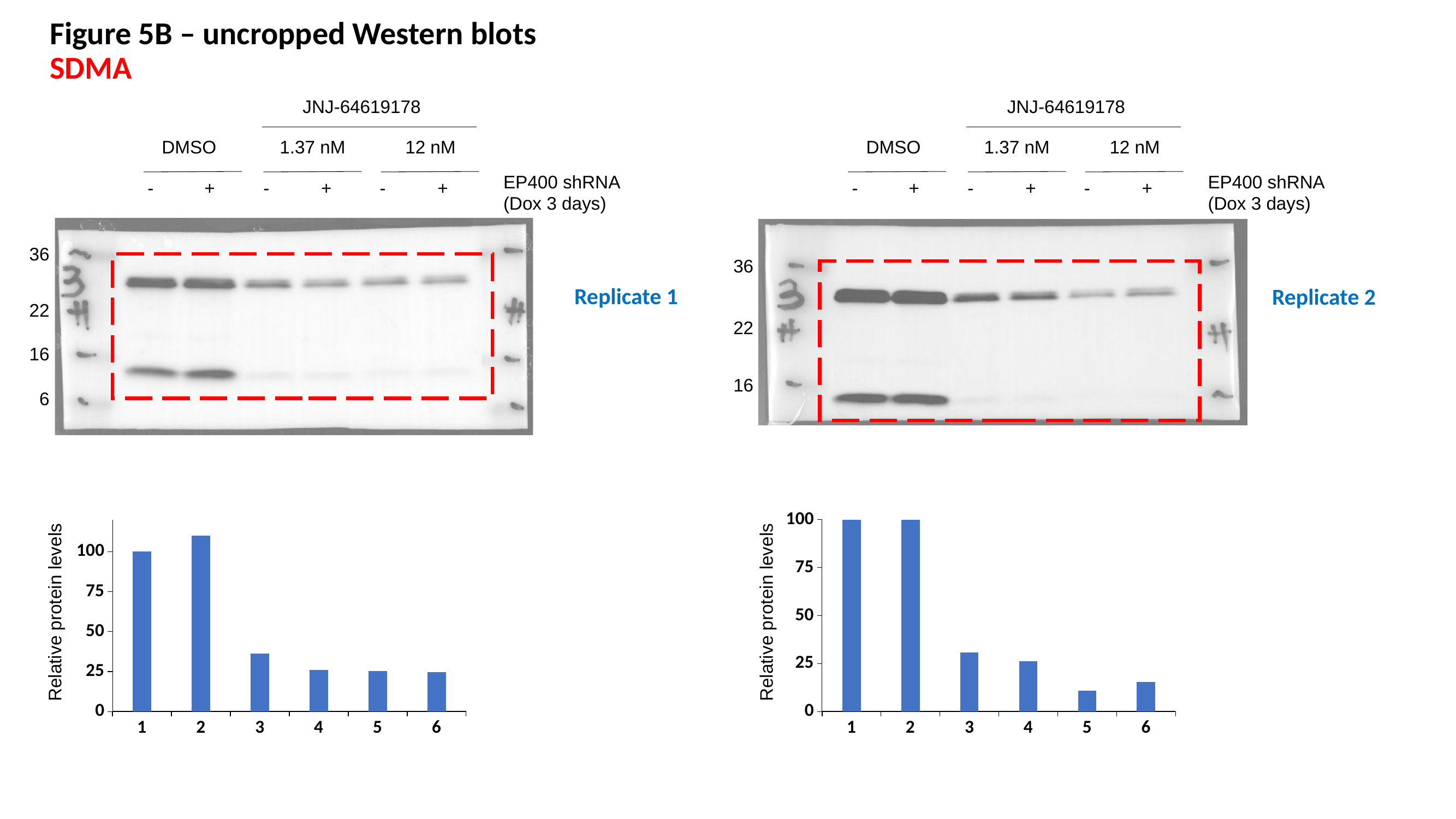

# Figure 5B – uncropped Western blots SDMA
JNJ-64619178
DMSO
1.37 nM
12 nM
EP400 shRNA
(Dox 3 days)
 -
 +
 -
 +
 -
 +
JNJ-64619178
DMSO
1.37 nM
12 nM
EP400 shRNA
(Dox 3 days)
 -
 +
 -
 +
 -
 +
36
36
Replicate 1
Replicate 2
22
22
16
16
6
### Chart
| Category | |
|---|---|
| 1 | 100.0 |
| 2 | 110.10317745952578 |
| 3 | 36.25628894339574 |
| 4 | 26.156963016025824 |
| 5 | 25.321925409992872 |
| 6 | 24.496555997492628 |
### Chart
| Category | |
|---|---|
| 1 | 100.0 |
| 2 | 103.85468984839716 |
| 3 | 30.747887337160478 |
| 4 | 26.203106891005334 |
| 5 | 10.906421832217337 |
| 6 | 15.52436135476023 |Relative protein levels
Relative protein levels

## Slide 23
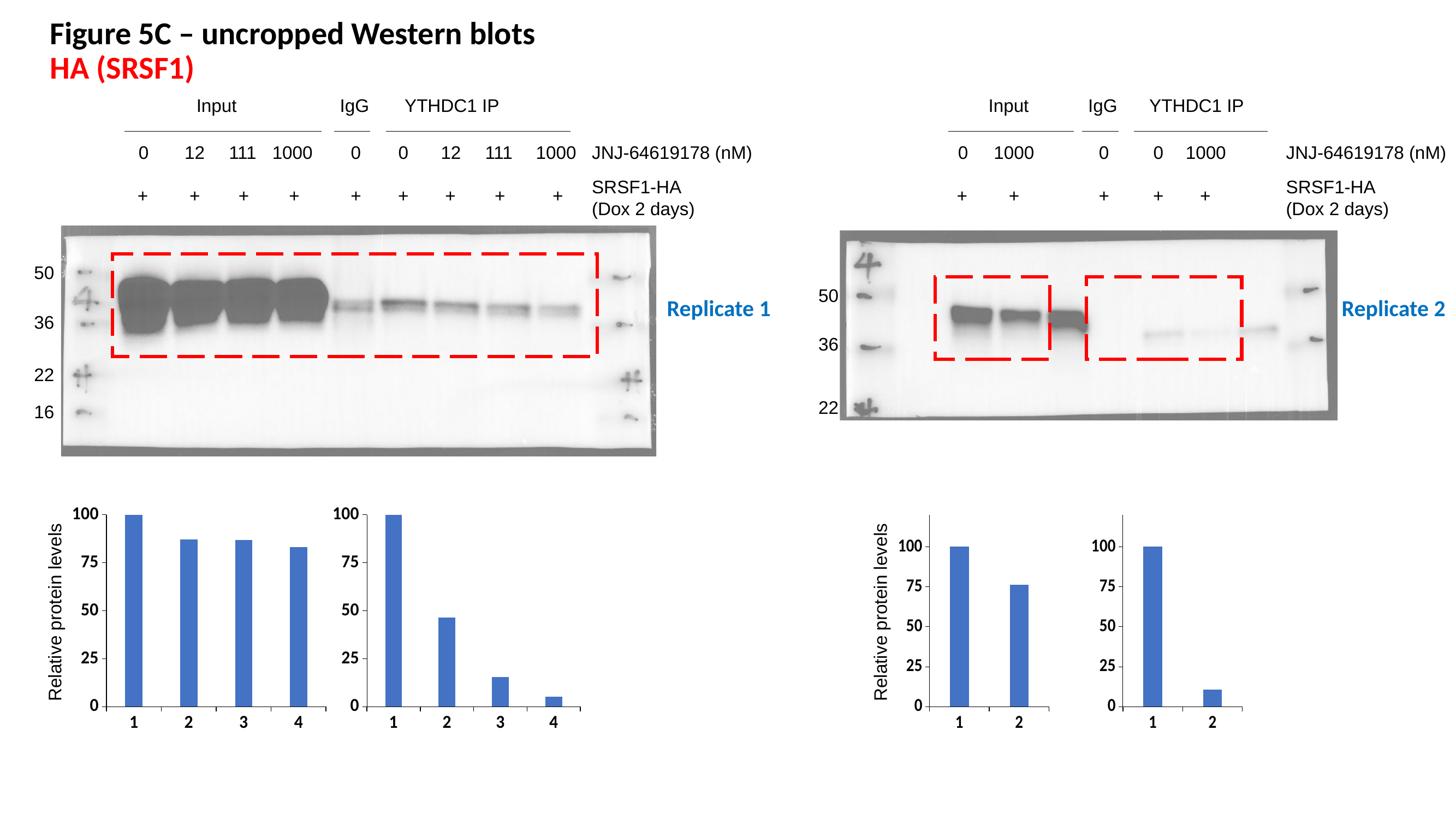

# Figure 5C – uncropped Western blots HA (SRSF1)
Input
IgG
YTHDC1 IP
0
12
111
1000
0
0
12
111
1000
JNJ-64619178 (nM)
SRSF1-HA
(Dox 2 days)
 +
 +
 +
 +
 +
 +
 +
 +
 +
Input
IgG
YTHDC1 IP
0
1000
0
0
1000
JNJ-64619178 (nM)
SRSF1-HA
(Dox 2 days)
 +
 +
 +
 +
 +
50
50
Replicate 1
Replicate 2
36
36
22
22
16
### Chart
| Category | |
|---|---|
| 1 | 100.0 |
| 2 | 87.26373327720978 |
| 3 | 87.00221674161128 |
| 4 | 83.18963594811012 |
### Chart
| Category | |
|---|---|
| 1 | 100.0 |
| 2 | 46.48176103576584 |
| 3 | 15.398366535059171 |
| 4 | 5.287078452227337 |
### Chart
| Category | |
|---|---|
| 1 | 100.0 |
| 2 | 76.0947775343932 |
### Chart
| Category | |
|---|---|
| 1 | 100.0 |
| 2 | 10.576324311903258 |Relative protein levels
Relative protein levels

## Slide 24
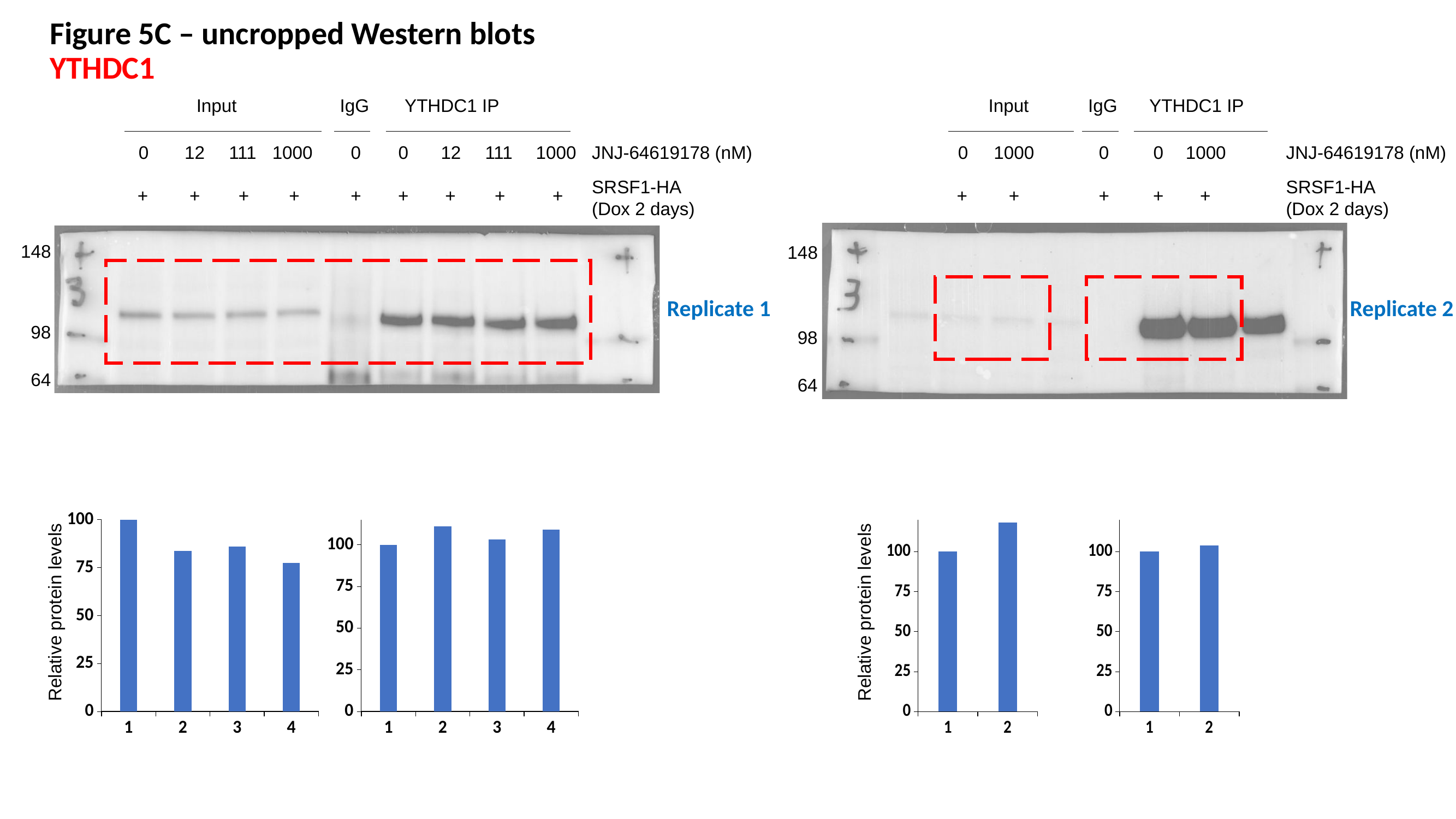

# Figure 5C – uncropped Western blots YTHDC1
Input
IgG
YTHDC1 IP
0
12
111
1000
0
0
12
111
1000
JNJ-64619178 (nM)
SRSF1-HA
(Dox 2 days)
 +
 +
 +
 +
 +
 +
 +
 +
 +
Input
IgG
YTHDC1 IP
0
1000
0
0
1000
JNJ-64619178 (nM)
SRSF1-HA
(Dox 2 days)
 +
 +
 +
 +
 +
148
148
Replicate 1
Replicate 2
98
98
64
64
### Chart
| Category | |
|---|---|
| 1 | 100.0 |
| 2 | 83.84155213371518 |
| 3 | 86.01938923461844 |
| 4 | 77.54997730855902 |
### Chart
| Category | |
|---|---|
| 1 | 100.0 |
| 2 | 111.12396721539162 |
| 3 | 103.11829269057372 |
| 4 | 108.91144306953875 |
### Chart
| Category | |
|---|---|
| 1 | 100.0 |
| 2 | 118.07290883293066 |
### Chart
| Category | |
|---|---|
| 1 | 100.0 |
| 2 | 104.05251924976359 |Relative protein levels
Relative protein levels

## Slide 25
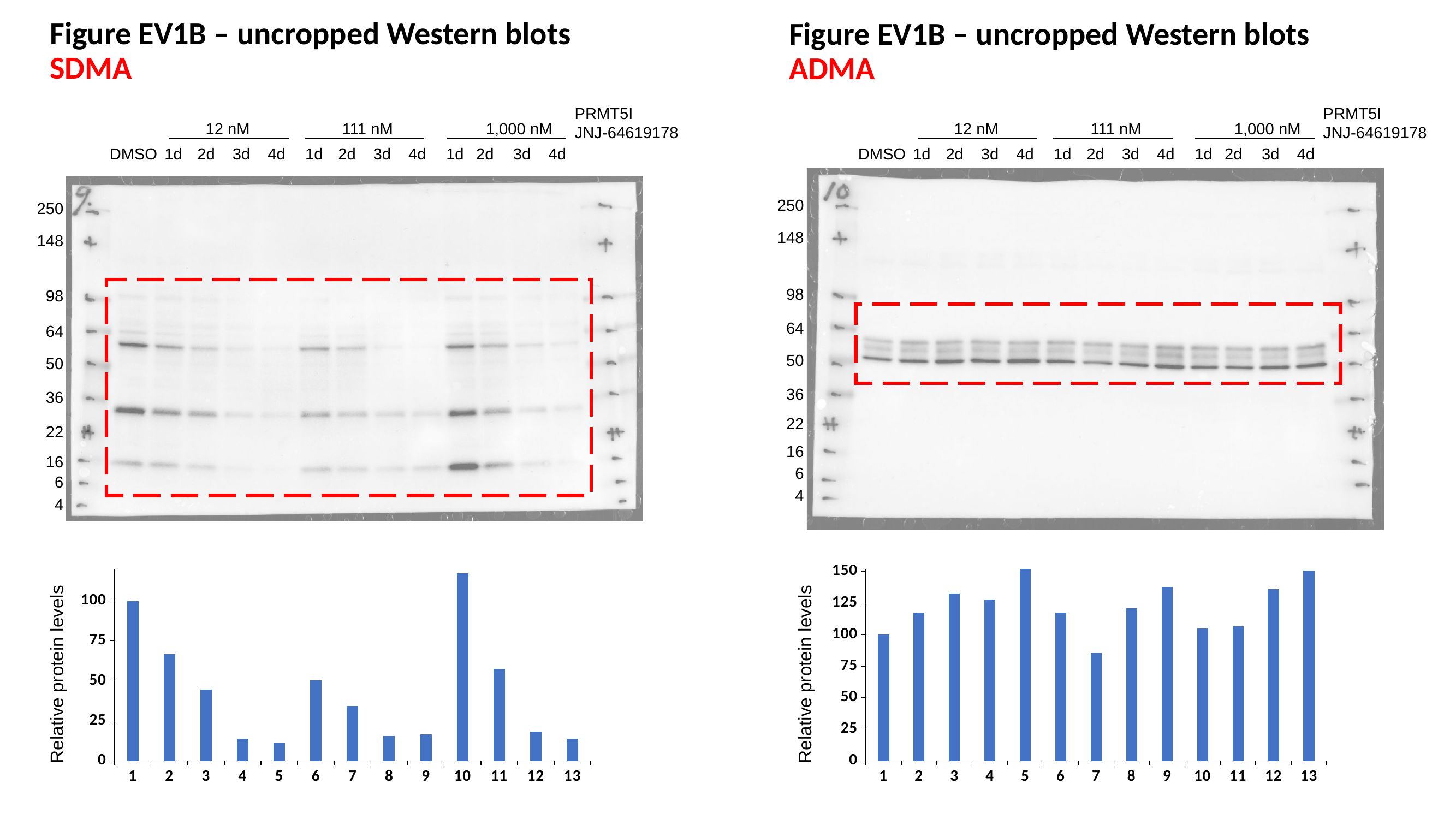

Figure EV1B – uncropped Western blots
ADMA
# Figure EV1B – uncropped Western blots SDMA
PRMT5I
JNJ-64619178
12 nM
111 nM
1,000 nM
DMSO
1d
2d
3d
4d
1d
2d
3d
4d
1d
2d
3d
4d
PRMT5I
JNJ-64619178
12 nM
111 nM
1,000 nM
DMSO
1d
2d
3d
4d
1d
2d
3d
4d
1d
2d
3d
4d
250
250
148
148
98
98
64
64
50
50
36
36
22
22
16
16
6
6
4
4
### Chart
| Category | |
|---|---|
| 1 | 100.0 |
| 2 | 66.7072896278464 |
| 3 | 44.57129932098049 |
| 4 | 13.789585791283908 |
| 5 | 11.533261044012376 |
| 6 | 50.406076167483896 |
| 7 | 34.45871665996059 |
| 8 | 15.68941393365295 |
| 9 | 16.53647769147009 |
| 10 | 117.32382887890176 |
| 11 | 57.61228145551593 |
| 12 | 18.272421624054612 |
| 13 | 13.807032978024502 |
### Chart
| Category | |
|---|---|
| 1 | 100.0 |
| 2 | 117.48685333313247 |
| 3 | 132.65648453090847 |
| 4 | 127.99138811216208 |
| 5 | 154.10152703876386 |
| 6 | 117.3750722567718 |
| 7 | 85.39769402074026 |
| 8 | 120.9779879701935 |
| 9 | 137.84873594385348 |
| 10 | 104.83185283364466 |
| 11 | 106.63374013018785 |
| 12 | 135.86878599682595 |
| 13 | 150.96852410188822 |Relative protein levels
Relative protein levels

## Slide 26
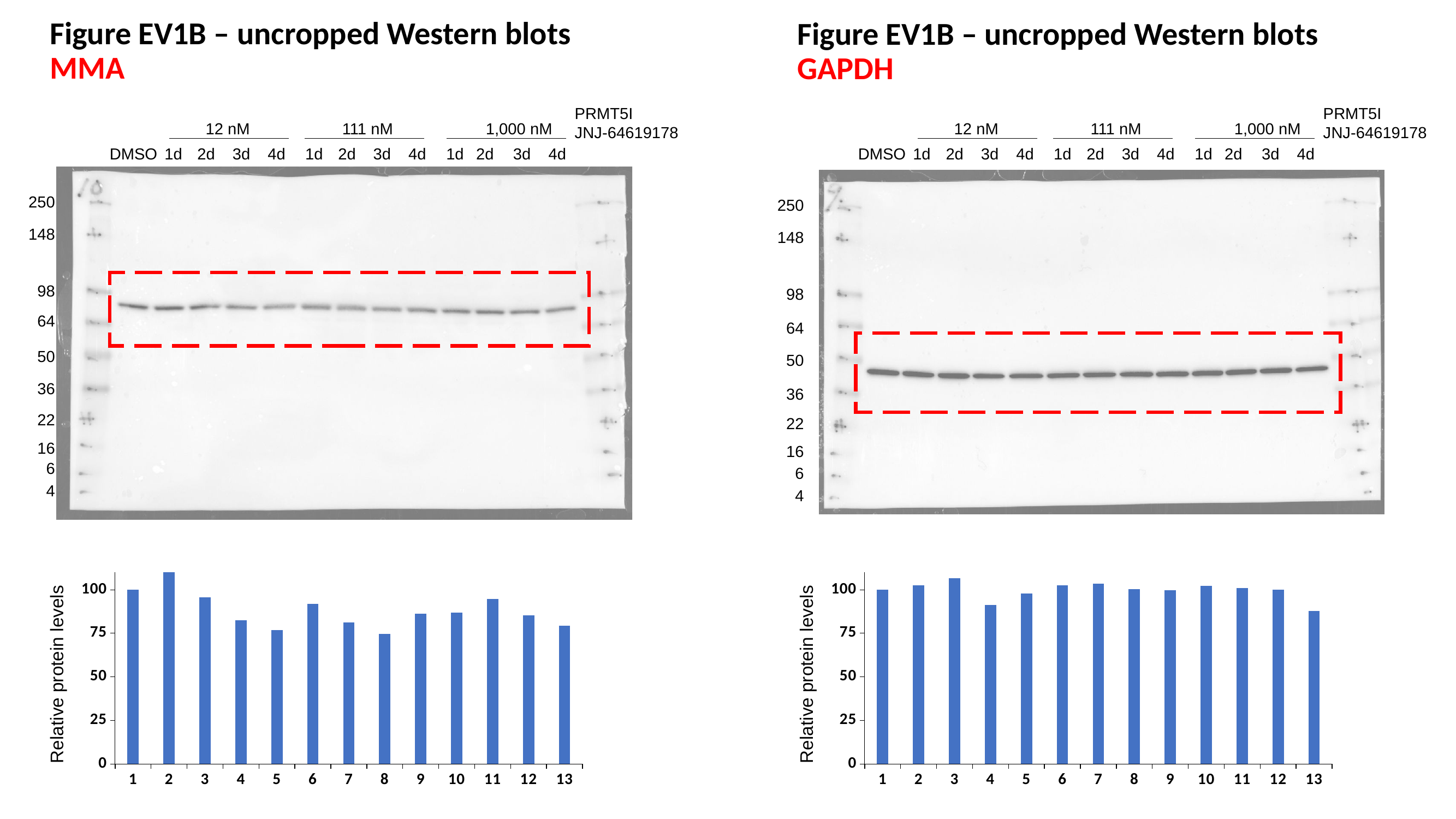

Figure EV1B – uncropped Western blots
GAPDH
# Figure EV1B – uncropped Western blots MMA
PRMT5I
JNJ-64619178
12 nM
111 nM
1,000 nM
DMSO
1d
2d
3d
4d
1d
2d
3d
4d
1d
2d
3d
4d
PRMT5I
JNJ-64619178
12 nM
111 nM
1,000 nM
DMSO
1d
2d
3d
4d
1d
2d
3d
4d
1d
2d
3d
4d
250
250
148
148
98
98
64
64
50
50
36
36
22
22
16
16
6
6
4
4
### Chart
| Category | |
|---|---|
| 1 | 100.0 |
| 2 | 110.42486649736647 |
| 3 | 95.5146116611986 |
| 4 | 82.50442839425396 |
| 5 | 76.8776964734479 |
| 6 | 91.80463428204646 |
| 7 | 81.36568803589496 |
| 8 | 74.75695798940993 |
| 9 | 86.31413935095028 |
| 10 | 86.89209385437178 |
| 11 | 94.70863225075493 |
| 12 | 85.32016931055757 |
| 13 | 79.46208074500376 |
### Chart
| Category | |
|---|---|
| 1 | 100.0 |
| 2 | 102.4731184886212 |
| 3 | 106.53568700921345 |
| 4 | 91.1255184993914 |
| 5 | 97.72713974604093 |
| 6 | 102.50190296324288 |
| 7 | 103.51802847441832 |
| 8 | 100.39163450939981 |
| 9 | 99.5882445888823 |
| 10 | 102.15379405235734 |
| 11 | 101.04978108165972 |
| 12 | 100.10556082865516 |
| 13 | 87.8003470702409 |Relative protein levels
Relative protein levels

## Slide 27
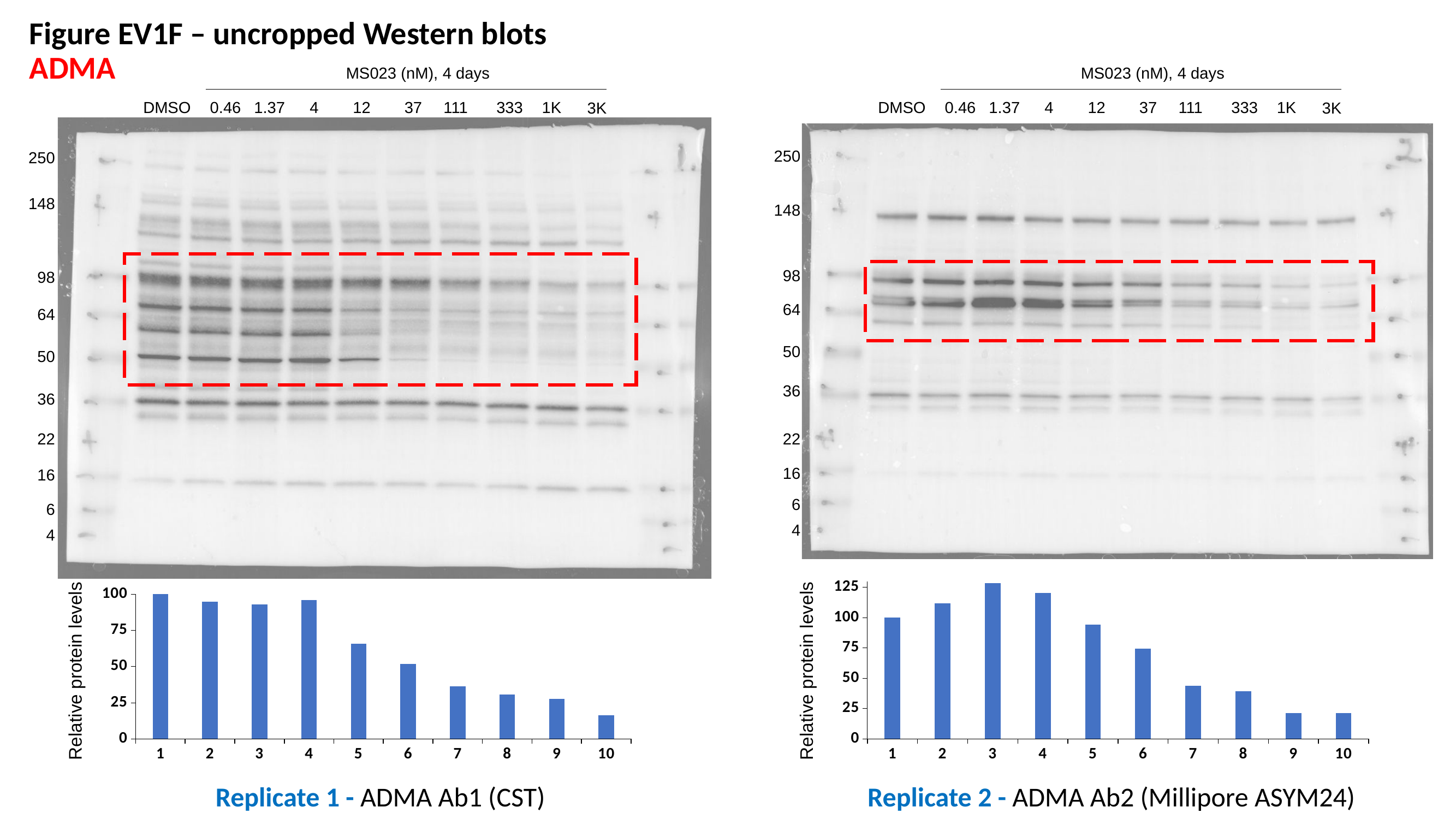

# Figure EV1F – uncropped Western blots ADMA
MS023 (nM), 4 days
DMSO
0.46
1.37
4
12
37
111
333
1K
3K
MS023 (nM), 4 days
DMSO
0.46
1.37
4
12
37
111
333
1K
3K
250
250
148
148
98
98
64
64
50
50
36
36
22
22
16
16
6
6
4
### Chart
| Category | |
|---|---|
| 1 | 100.0 |
| 2 | 112.1082462762384 |
| 3 | 128.5115222162066 |
| 4 | 120.26850819234846 |
| 5 | 94.45275402279361 |
| 6 | 74.4423426124332 |
| 7 | 44.041152490951966 |
| 8 | 39.25244187524189 |
| 9 | 21.261451481502842 |
| 10 | 21.521647048832325 |4
### Chart
| Category | |
|---|---|
| 1 | 100.0 |
| 2 | 94.77543713957188 |
| 3 | 93.0266320489762 |
| 4 | 95.95494203471748 |
| 5 | 65.95982363370896 |
| 6 | 51.802371694640094 |
| 7 | 36.35586187935839 |
| 8 | 30.583822280290647 |
| 9 | 27.521742280665677 |
| 10 | 16.440487751777898 |Relative protein levels
Relative protein levels
Replicate 1 - ADMA Ab1 (CST)
Replicate 2 - ADMA Ab2 (Millipore ASYM24)

## Slide 28
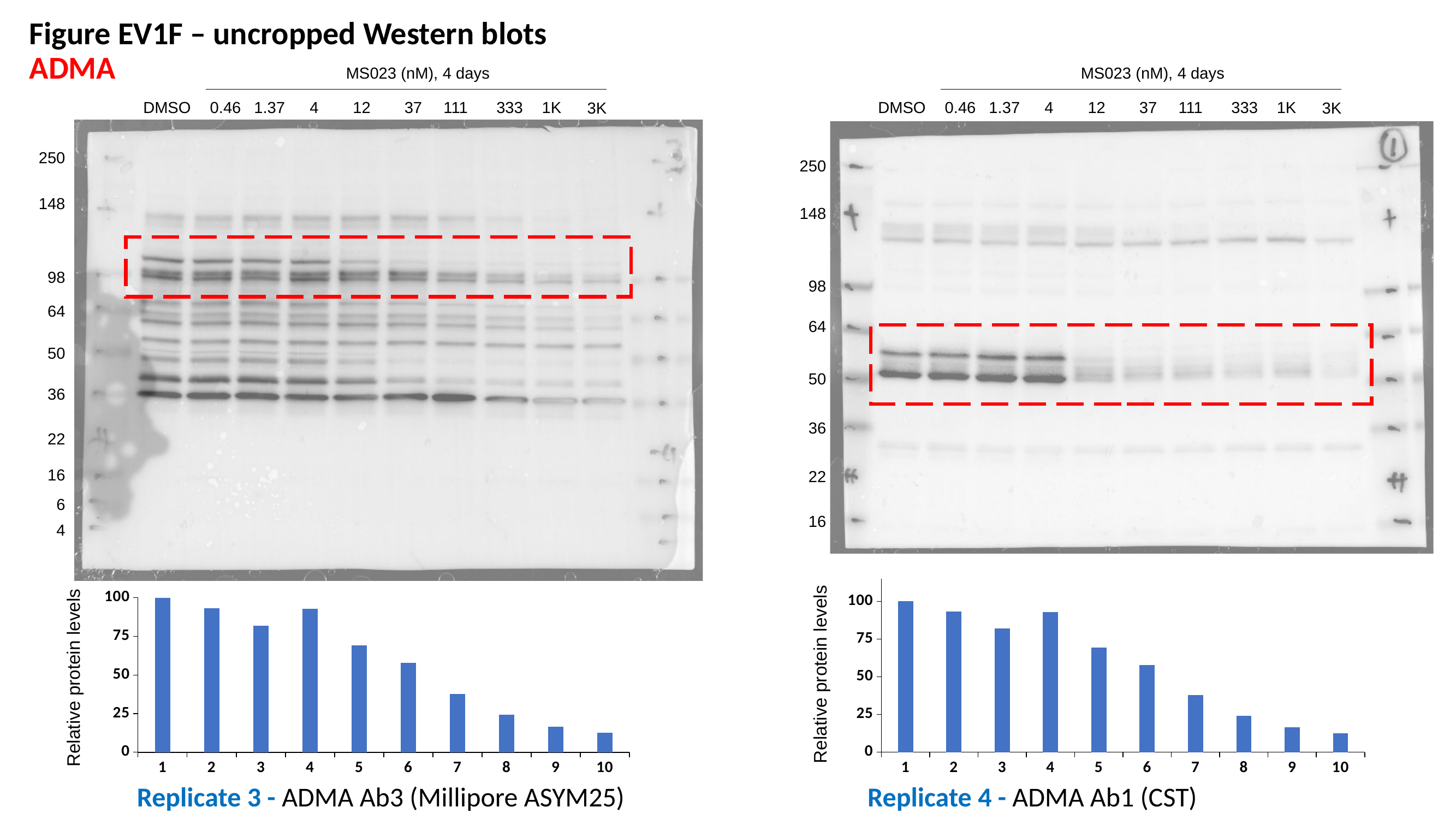

# Figure EV1F – uncropped Western blots ADMA
MS023 (nM), 4 days
DMSO
0.46
1.37
4
12
37
111
333
1K
3K
MS023 (nM), 4 days
DMSO
0.46
1.37
4
12
37
111
333
1K
3K
250
250
148
148
98
98
64
64
50
50
36
36
22
16
22
6
### Chart
| Category | |
|---|---|
| 1 | 100.0 |
| 2 | 93.29085401395153 |
| 3 | 81.91626666380152 |
| 4 | 92.76388101716677 |
| 5 | 69.27636862759846 |
| 6 | 57.80278224712523 |
| 7 | 37.83634488260464 |
| 8 | 24.21324226608898 |
| 9 | 16.35857828192614 |
| 10 | 12.487835391322333 |16
4
### Chart
| Category | |
|---|---|
| 1 | 100.0 |
| 2 | 93.29085401395153 |
| 3 | 81.91626666380152 |
| 4 | 92.76388101716677 |
| 5 | 69.27636862759846 |
| 6 | 57.80278224712523 |
| 7 | 37.83634488260464 |
| 8 | 24.21324226608898 |
| 9 | 16.35857828192614 |
| 10 | 12.487835391322333 |Relative protein levels
Relative protein levels
Replicate 3 - ADMA Ab3 (Millipore ASYM25)
Replicate 4 - ADMA Ab1 (CST)

## Slide 29
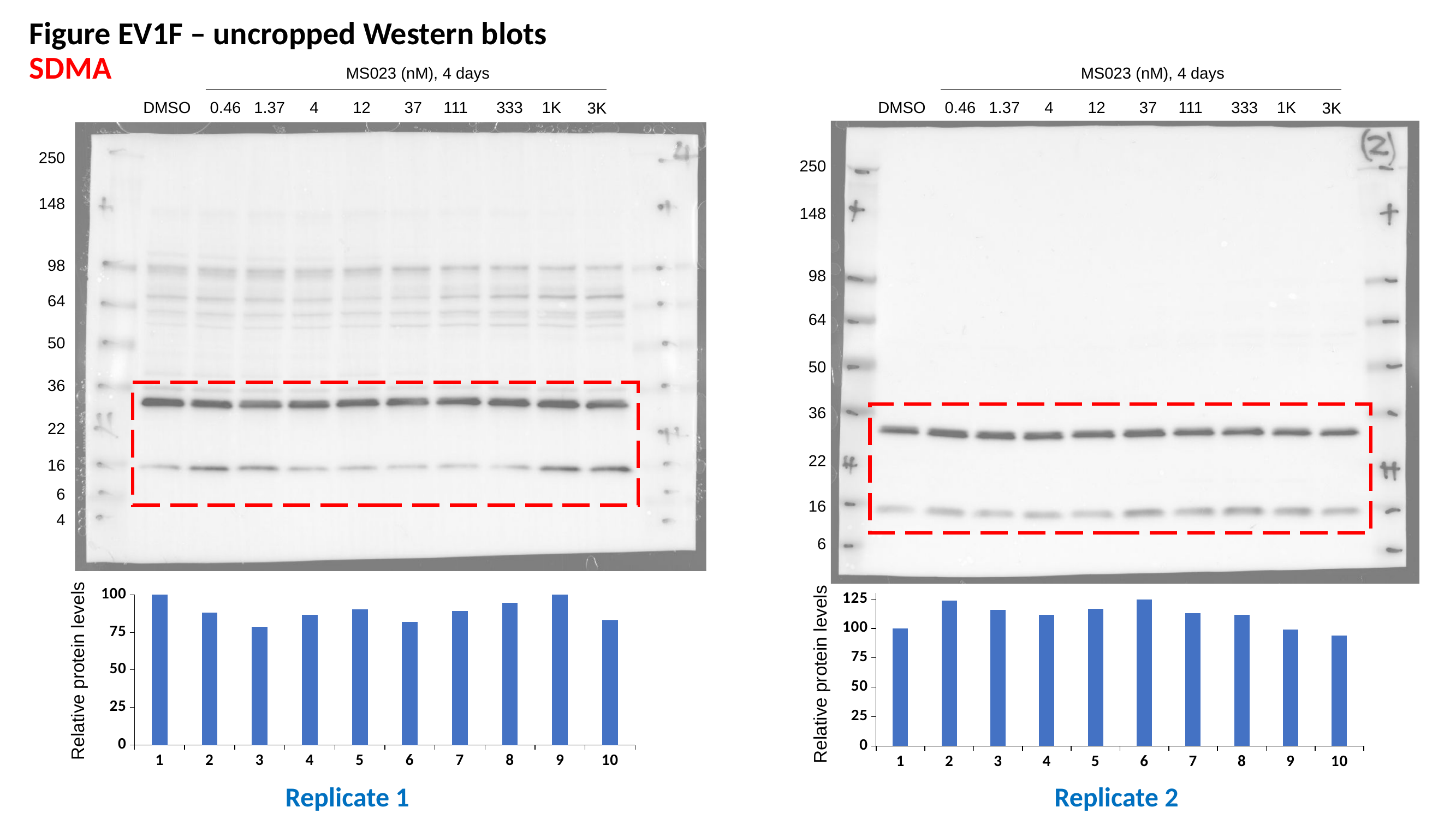

# Figure EV1F – uncropped Western blots SDMA
MS023 (nM), 4 days
DMSO
0.46
1.37
4
12
37
111
333
1K
3K
MS023 (nM), 4 days
DMSO
0.46
1.37
4
12
37
111
333
1K
3K
250
250
148
148
98
98
64
64
50
50
36
36
22
22
16
6
16
4
### Chart
| Category | |
|---|---|
| 1 | 100.0 |
| 2 | 123.44288540773756 |
| 3 | 115.60012809228768 |
| 4 | 111.61736148024633 |
| 5 | 116.62935078858374 |
| 6 | 124.40453434717817 |
| 7 | 112.9182852572649 |
| 8 | 111.4276025235412 |
| 9 | 99.2384432610486 |
| 10 | 93.7560833644842 |6
### Chart
| Category | |
|---|---|
| 1 | 100.0 |
| 2 | 88.13920421625552 |
| 3 | 78.65521259977022 |
| 4 | 86.75198010877457 |
| 5 | 90.17322564275703 |
| 6 | 81.99718518296034 |
| 7 | 89.27123703316826 |
| 8 | 94.57968186127722 |
| 9 | 100.28185251374731 |
| 10 | 83.1638639936473 |Relative protein levels
Relative protein levels
Replicate 1
Replicate 2

## Slide 30
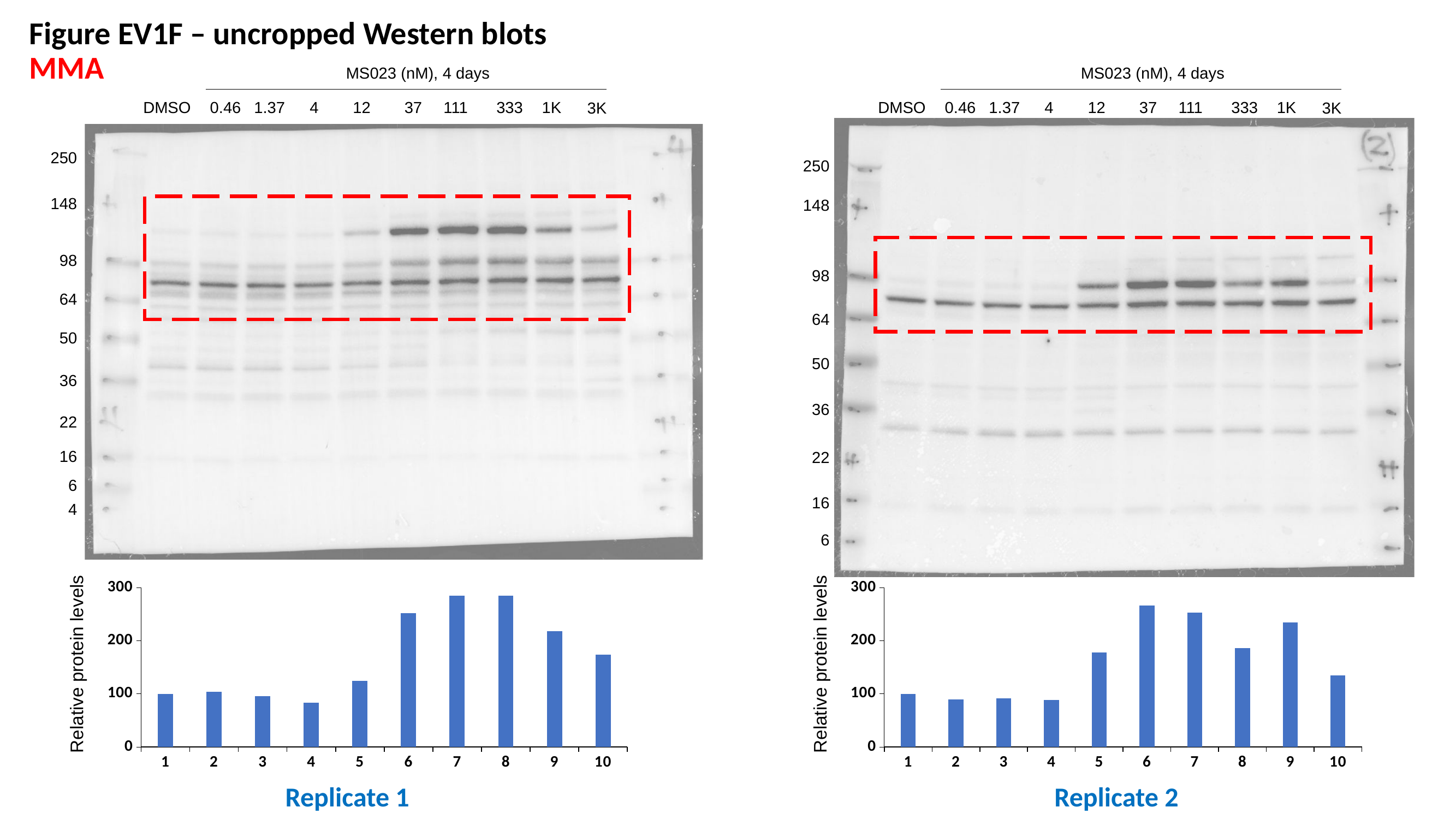

# Figure EV1F – uncropped Western blots MMA
MS023 (nM), 4 days
DMSO
0.46
1.37
4
12
37
111
333
1K
3K
MS023 (nM), 4 days
DMSO
0.46
1.37
4
12
37
111
333
1K
3K
250
250
148
148
98
98
64
64
50
50
36
36
22
16
22
6
16
4
### Chart
| Category | |
|---|---|
| 1 | 100.0 |
| 2 | 103.51500126446862 |
| 3 | 95.3762850074426 |
| 4 | 83.12475092777211 |
| 5 | 124.52320459231665 |
| 6 | 252.17421082521244 |
| 7 | 285.35422351419004 |
| 8 | 284.62830726597906 |
| 9 | 217.7235179169422 |
| 10 | 173.58406253183455 |
### Chart
| Category | |
|---|---|
| 1 | 100.0 |
| 2 | 89.65211468861716 |
| 3 | 91.27949419066103 |
| 4 | 88.62766551787648 |
| 5 | 177.4382600949533 |
| 6 | 266.7800778735946 |
| 7 | 253.25625583962815 |
| 8 | 185.71692161383254 |
| 9 | 234.09823702627128 |
| 10 | 134.80189070645912 |6
Relative protein levels
Relative protein levels
Replicate 1
Replicate 2

## Slide 31
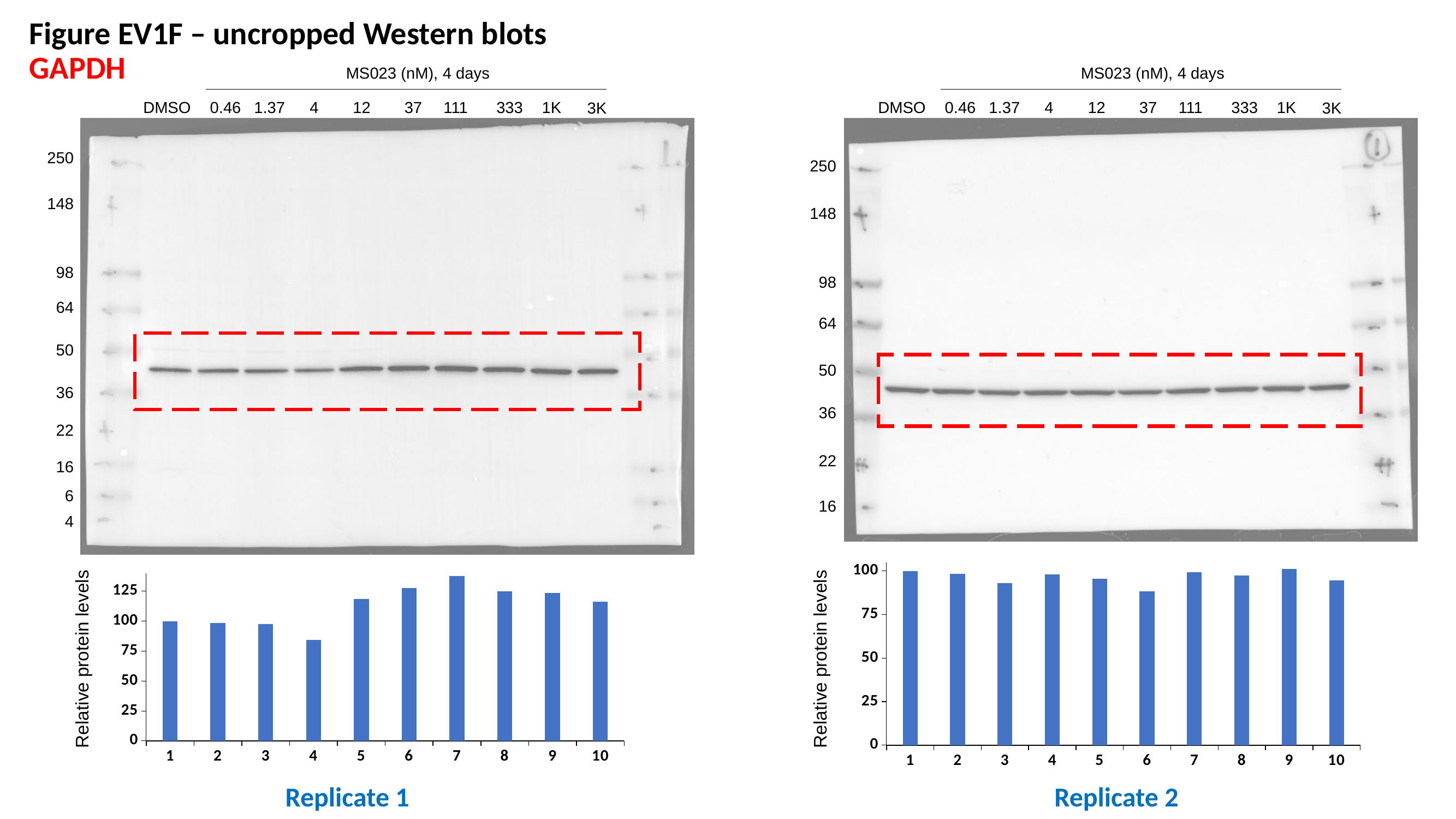

# Figure EV1F – uncropped Western blots GAPDH
MS023 (nM), 4 days
DMSO
0.46
1.37
4
12
37
111
333
1K
3K
MS023 (nM), 4 days
DMSO
0.46
1.37
4
12
37
111
333
1K
3K
250
250
148
148
98
98
64
64
50
50
36
36
22
22
16
6
### Chart
| Category | |
|---|---|
| 1 | 100.0 |
| 2 | 98.51650321005485 |
| 3 | 93.11117760181202 |
| 4 | 97.92895670207297 |
| 5 | 95.60465860827962 |
| 6 | 88.42860297997466 |
| 7 | 99.28108395823688 |
| 8 | 97.48119186450721 |
| 9 | 101.15078029165043 |
| 10 | 94.71738437312015 |16
### Chart
| Category | |
|---|---|
| 1 | 100.0 |
| 2 | 98.66019585827152 |
| 3 | 97.53395331877287 |
| 4 | 84.41285009550539 |
| 5 | 118.352159192888 |
| 6 | 127.6757808701964 |
| 7 | 137.62566942129405 |
| 8 | 124.87169704979226 |
| 9 | 123.35238856909324 |
| 10 | 116.36512738222231 |4
Relative protein levels
Relative protein levels
Replicate 1
Replicate 2

## Slide 32
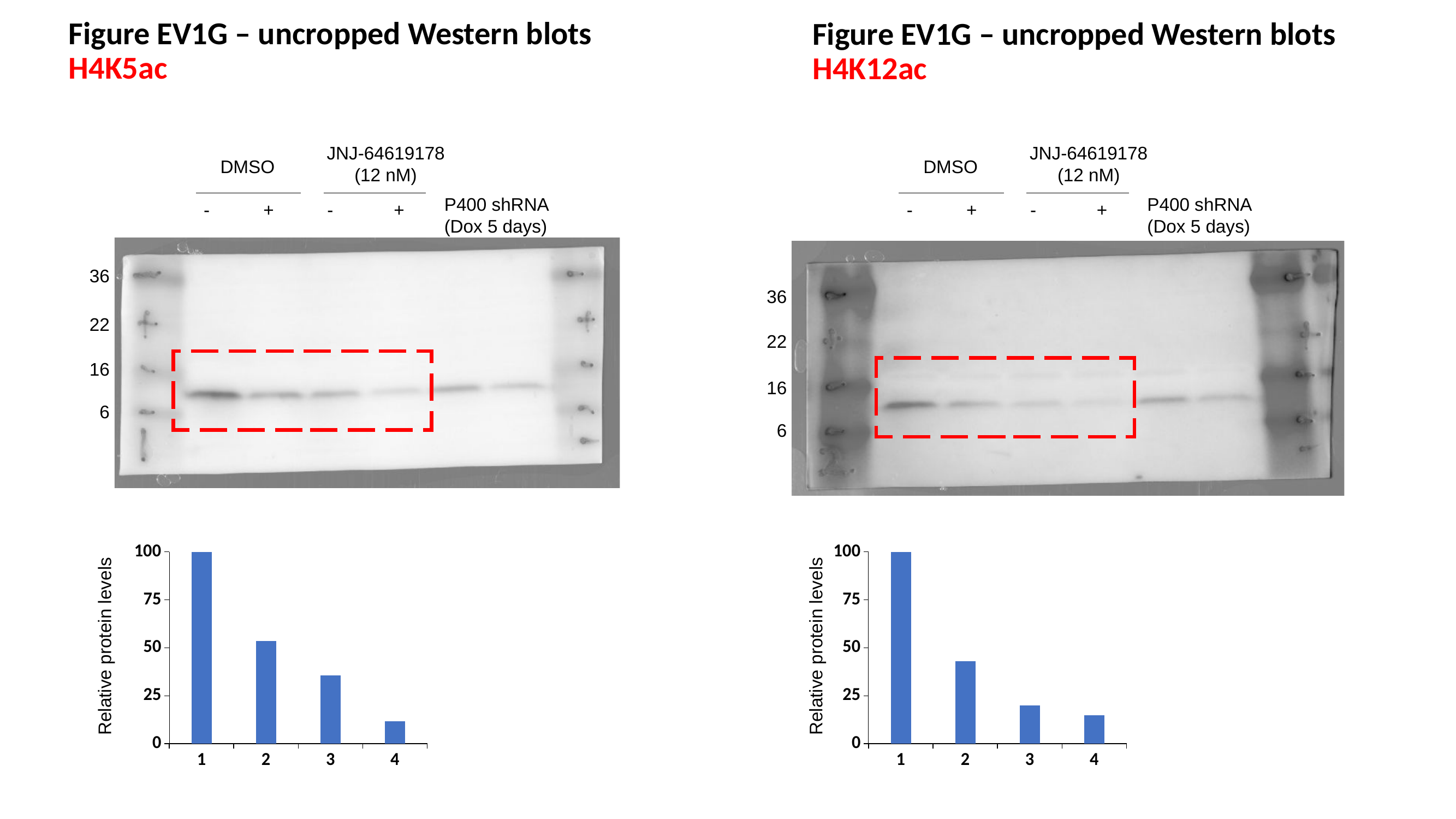

Figure EV1G – uncropped Western blots
H4K12ac
# Figure EV1G – uncropped Western blots H4K5ac
JNJ-64619178
(12 nM)
DMSO
P400 shRNA
(Dox 5 days)
 -
 +
 -
 +
JNJ-64619178
(12 nM)
DMSO
P400 shRNA
(Dox 5 days)
 -
 +
 -
 +
36
36
22
22
16
16
6
6
### Chart
| Category | |
|---|---|
| 1 | 100.0 |
| 2 | 53.51817829386821 |
| 3 | 35.48538481579172 |
| 4 | 11.659866078801095 |
### Chart
| Category | |
|---|---|
| 1 | 100.0 |
| 2 | 42.95012884786079 |
| 3 | 19.848410409501913 |
| 4 | 14.727476193052969 |Relative protein levels
Relative protein levels

## Slide 33
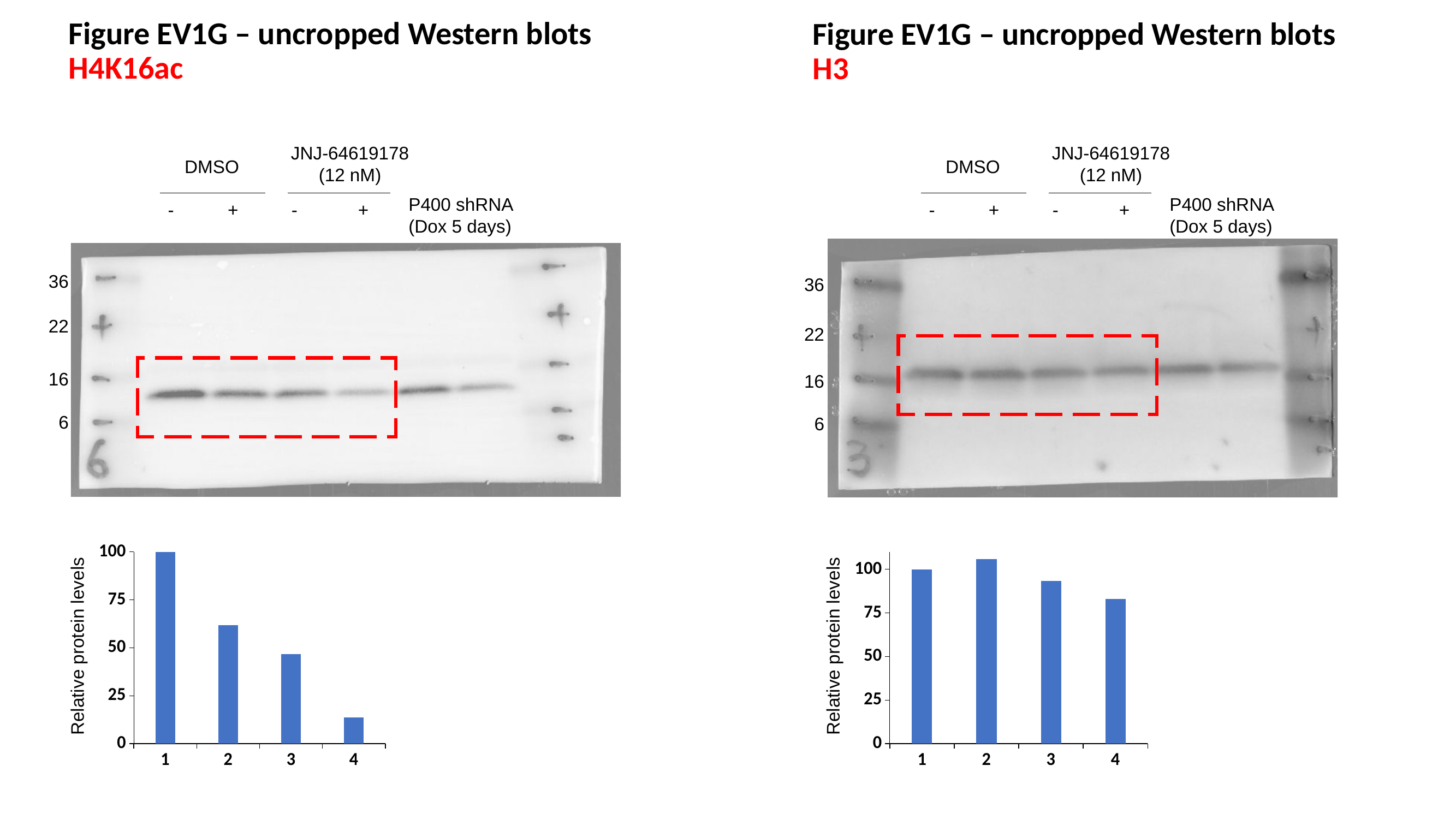

Figure EV1G – uncropped Western blots
H3
# Figure EV1G – uncropped Western blots H4K16ac
JNJ-64619178
(12 nM)
DMSO
P400 shRNA
(Dox 5 days)
 -
 +
 -
 +
JNJ-64619178
(12 nM)
DMSO
P400 shRNA
(Dox 5 days)
 -
 +
 -
 +
36
36
22
22
16
16
6
6
### Chart
| Category | |
|---|---|
| 1 | 100.0 |
| 2 | 61.79941039486122 |
| 3 | 46.666848324996664 |
| 4 | 13.638360376685062 |
### Chart
| Category | |
|---|---|
| 1 | 100.0 |
| 2 | 105.80402291166496 |
| 3 | 93.2228275361131 |
| 4 | 82.95593662733125 |Relative protein levels
Relative protein levels

## Slide 34
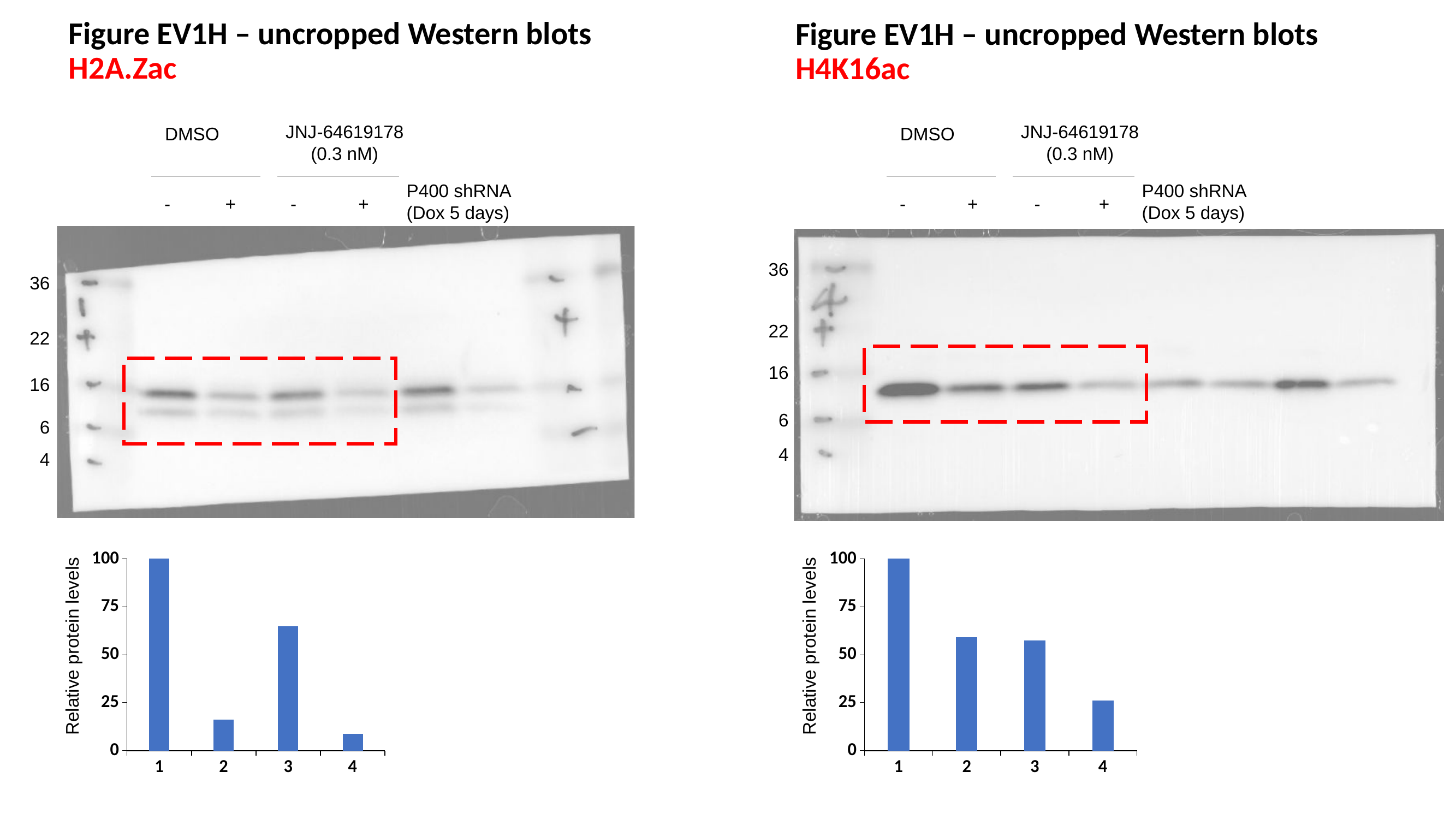

Figure EV1H – uncropped Western blots
H4K16ac
# Figure EV1H – uncropped Western blots H2A.Zac
JNJ-64619178
(0.3 nM)
DMSO
 -
 +
 -
 +
P400 shRNA (Dox 5 days)
JNJ-64619178
(0.3 nM)
DMSO
 -
 +
 -
 +
P400 shRNA (Dox 5 days)
36
36
22
22
16
16
6
6
4
4
### Chart
| Category | |
|---|---|
| 1 | 100.0 |
| 2 | 16.09563110954297 |
| 3 | 64.752908310712 |
| 4 | 8.723083849493902 |
### Chart
| Category | |
|---|---|
| 1 | 100.0 |
| 2 | 59.096651237397104 |
| 3 | 57.351727986855394 |
| 4 | 26.03841658084076 |Relative protein levels
Relative protein levels

## Slide 35
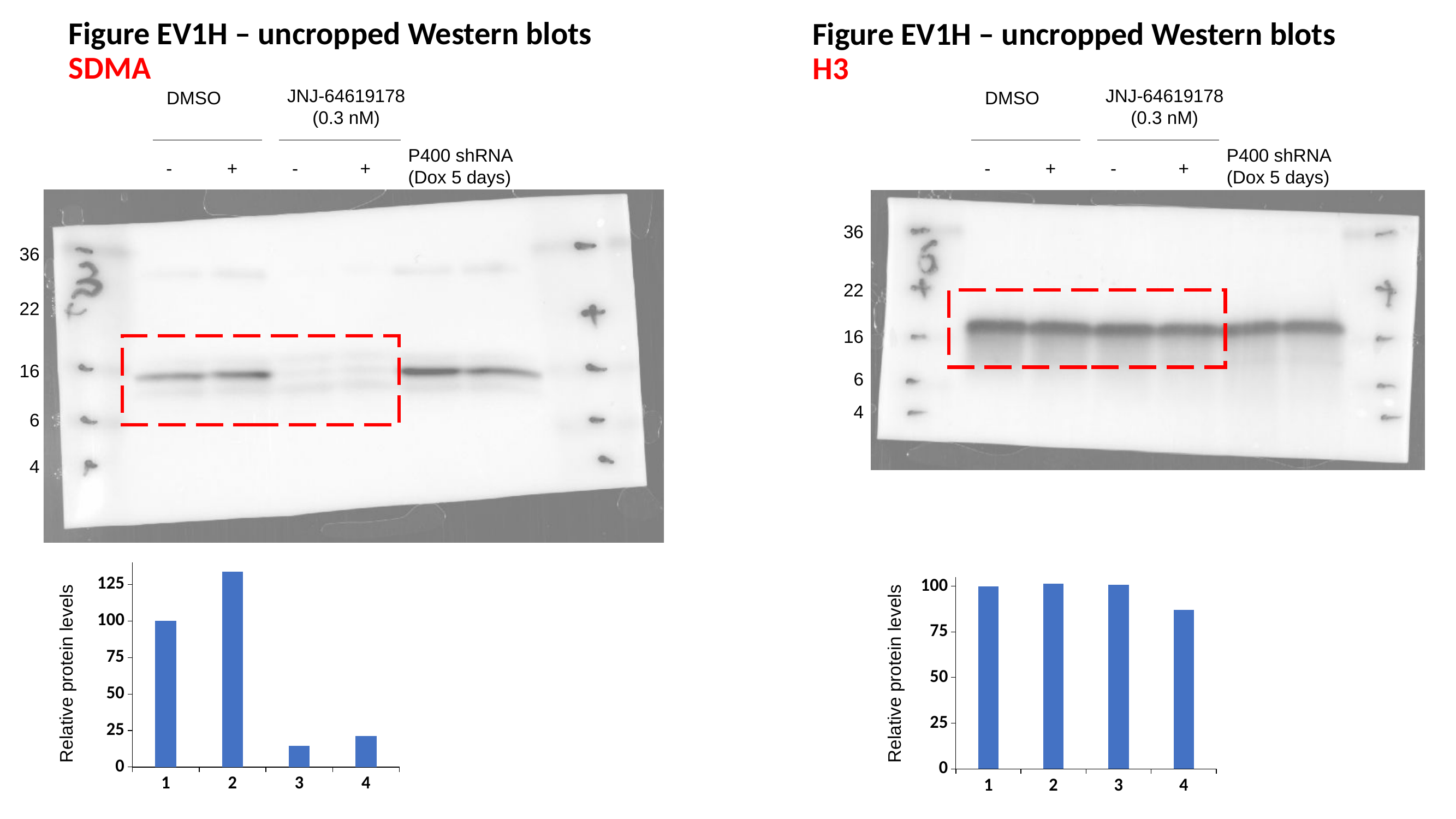

Figure EV1H – uncropped Western blots
H3
# Figure EV1H – uncropped Western blots SDMA
JNJ-64619178
(0.3 nM)
DMSO
 -
 +
 -
 +
P400 shRNA (Dox 5 days)
JNJ-64619178
(0.3 nM)
DMSO
 -
 +
 -
 +
P400 shRNA (Dox 5 days)
36
36
22
22
16
16
6
4
6
4
### Chart
| Category | |
|---|---|
| 1 | 100.0 |
| 2 | 133.60203753357334 |
| 3 | 14.395495072163758 |
| 4 | 21.135182310798342 |
### Chart
| Category | |
|---|---|
| 1 | 100.0 |
| 2 | 101.23731403109714 |
| 3 | 100.69743432939956 |
| 4 | 86.94070762290718 |Relative protein levels
Relative protein levels
